# Supplementary material for: Concentration-Dependent Global Quantitative Proteome Response of Staphylococcus epidermidis RP62A Biofilms to Subinhibitory Tigecycline
Source: Cells. 2022 Nov 3;11(21):3488. doi: 10.3390/cells11213488 (PMC9655631; doi:10.3390/cells11213488)
Supplement: Supplementary file 1 [file cells-11-03488-s001.zip › cells-1910341-supplementary.pdf]

Table. S1. Identified proteins from tigecycline-treated *S. epidermidis* strain RP62A biofilms

Locus tag	Protein	Gene	PATRIC ID	COG	Description	Fold ratio		
						T1	T2	T3
SEA0001	Q5HS67	SEA0001	fig|176279.9.peg.2471	S	Replication-associated protein	3.72	1.56	8.83
SEA0010	Q5HS59	aphA	fig|176279.9.peg.2480	J	Aminoglycoside 3'-phosphotransferase	-5.27	-1000.00	1.94
SEA0017	Q5HS52	SEA0017	fig|176279.9.peg.2486	V	ABC transporter, permease protein	0.00	0.00	1000.00
SEA0027	Q5HS44	SEA0027	fig|176279.9.peg.2497	S	Mobilization protein	1000.00	1000.00	1000.00
SEA0030	Q5HS41	SEA0030	fig|176279.9.peg.2499	-	Mobilization protein	1000.00	1000.00	1000.00
SEA0033	Q5HS39	repA	fig|176279.9.peg.2503	S	Replication-associated protein RepA	0.00	0.00	1000.00
SERP0006	Q5HS33	SERP0006	fig|176279.9.peg.6	K	Chromosome partioning protein, ParB family	0.00	1000.00	1000.00
SERP0013	Q5HS26	SERP0013	fig|176279.9.peg.12	S	Yip1 domain-containing protein	-1000.00	84.20	210.00
SERP0014	Q5HS25	SERP0014	fig|176279.9.peg.13	M	RND family efflux transporter, MFP component	9.44	28.40	27.71
SERP0015	Q5HS24	SERP0015	fig|176279.9.peg.14	V	ABC transporter, ATP-binding protein	0.00	0.00	1000.00
SERP0016	Q5HS23	SERP0016	fig|176279.9.peg.15	V	ABC transporter, permease protein	0.00	0.00	1000.00
SERP0022	Q5HS17	SERP0022	fig|176279.9.peg.20	-	Uncharacterized protein	-1000.00	1.48	-1000.00
SERP0024	Q5HS15	SERP0024	fig|176279.9.peg.23	U	Signal peptidase I	0.00	1000.00	1000.00
SERP0032	Q5HS07	SERP0032	fig|176279.9.peg.31	I	Probable acetyl-CoA acyltransferase	-8.11	1.22	1.61
SERP0038	Q5HS01	SERP0038	fig|176279.9.peg.37	K	SpoOJ protein	-1000.00	-1000.00	-1000.00
SERP0041	Q5HRZ8	ychF	fig|176279.9.peg.40	J	Ribosome-binding ATPase YchF	-1000.00	-1000.00	2.82
SERP0045	Q5HRZ4	ssb	fig|176279.9.peg.44	L	Single-stranded DNA-binding protein	-1000.00	-37.88	-27.62
SERP0046	Q5HRZ3	rpsR	fig|176279.9.peg.45	J	30S ribosomal protein S18	0.00	0.00	1000.00
SERP0057	Q5HRY4	SERP0057	fig|176279.9.peg.52	-	Uncharacterized protein	6.48	2.04	7.14
SERP0059	Q5HRY2	ahpF	fig|176279.9.peg.53	O	Alkyl hydroperoxide reductase subunit F	-1000.00	-17.64	2.11
SERP0060	Q5HRY1	ahpC	fig|176279.9.peg.54	O	Alkyl hydroperoxide reductase C	2.83	2.38	1.54
SERP0063	Q5HRX8	SERP0063	fig|176279.9.peg.57	U	Sodium:dicarboxylate symporter family protein	-1000.00	-1000.00	-1000.00
SERP0066	Q5HRX5	SERP0066	fig|176279.9.peg.60	S	UPF0355 protein SERP0066	1.43	-1.46	-2.40

SERP0067	Q5HRX4	xpt	fig|176279.9.peg.61	F	Xanthine phosphoribosyltransferase	-1.42	2.63	3.13
SERP0069	Q5HRX2	guaB	fig|176279.9.peg.63	F	Inosine-5'-monophosphate dehydrogenase	-8.04	-2.66	1.17
SERP0070	Q5HRX1	guaA	fig|176279.9.peg.64	F	GMP synthase [glutamine-hydrolyzing]	-1000.00	-1000.00	9.91
SERP0073	Q5HRW9	SERP0073	fig|176279.9.peg.69	-	Uncharacterized protein	1000.00	1000.00	1000.00
SERP0074	Q5HRW8	SERP0074	fig|176279.9.peg.70	-	DUF4352 domain-containing protein	2.31	2.74	3.49
SERP0085	Q5HRV7	SERP0085	fig|176279.9.peg.80	S	UPF0753 protein SERP0085	1000.00	0.00	1000.00
SERP0091	Q5HRV1	SERP0091	fig|176279.9.peg.85	-	Uncharacterized protein	1000.00	1000.00	0.00
SERP0099	Q5HRU3	SERP0099	fig|176279.9.peg.92	P	Lipoprotein	2.10	3.66	6.04
SERP0109	Q5HRT4	gltD	fig|176279.9.peg.102	C	Glutamate synthase, small subunit	0.00	0.00	1000.00
SERP0115	Q5HRS8	SERP0115	fig|176279.9.peg.108	S	Nucleoid-associated protein SERP0115	1.64	-1.94	-2.02
SERP0120	Q5HRS3	tmk	fig|176279.9.peg.112	F	Thymidylate kinase	-1000.00	-1000.00	-1000.00
SERP0121	Q5HRS2	SERP0121	fig|176279.9.peg.113	S	Uncharacterized protein	-2.19	-1000.00	-2.00
SERP0122	Q5HRS1	SERP0122	fig|176279.9.peg.114	L	DNA polymerase III, delta prime subunit, putative	-1000.00	-1000.00	-1000.00
SERP0124	Q5HRR9	SERP0124	fig|176279.9.peg.116	L	Initiation-control protein YabA	-1.29	-1.24	-6.85
SERP0128	Q5HRR5	metG	fig|176279.9.peg.120	J	Methionine--tRNA ligase	0.00	1000.00	1000.00
SERP0129	Q5HRR4	SERP0129	fig|176279.9.peg.121	L	Deoxyribonuclease, TatD family	0.00	0.00	1000.00
SERP0135	Q5HRQ8	SERP0135	fig|176279.9.peg.127	J	Endoribonuclease L-PSP, putative	-1000.00	-1.23	-1000.00
SERP0136	Q5HRQ7	spoVG	fig|176279.9.peg.128	D	Putative septation protein SpoVG	2.05	1.30	2.86
SERP0139	Q5HRQ4	rplY	fig|176279.9.peg.131	J	50S ribosomal protein L25	-2.10	2.98	8.57
SERP0141	Q5HRQ2	mfd	fig|176279.9.peg.133	L	Transcription-repair-coupling factor	-1000.00	-1000.00	-1000.00
SERP0144	Q5HRP9	SERP0144	fig|176279.9.peg.136	J	S4 domain protein	1000.00	0.00	0.00
SERP0145	Q5HRP8	SERP0145	fig|176279.9.peg.137	D	Cell-division protein DivIC, putative	1000.00	1000.00	1000.00
SERP0146	Q5HRP7	SERP0146	fig|176279.9.peg.138	J	General stress protein 13	14.60	26.70	30.04
SERP0149	Q5HRP4	hpt	fig|176279.9.peg.140	F	Hypoxanthine-guanine phosphoribosyltransferase	-4.04	1.27	1.97
SERP0150	Q5HRP3	ftsH	fig|176279.9.peg.141	O	ATP-dependent zinc metalloprotease FtsH	2.51	3.53	4.63
SERP0152	Q5HRP1	cysK	fig|176279.9.peg.143	E	Cysteine synthase	-4.78	-1.17	-1.41

SERP0158	Q5HRN5	pdxS	fig|176279.9.peg.149	H	Pyridoxal 5'-phosphate synthase subunit PdxS	-2.28	-1.23	-1.63
SERP0163	Q5HRN0	SERP0163	fig|176279.9.peg.154	S	UvrB/UvrC domain protein	-1000.00	2.19	1.63
SERP0165	Q5HRM8	clpC	fig|176279.9.peg.156	O	ATP-dependent Clp protease ATP-binding subunit ClpC	6.85	4.58	5.69
SERP0168	Q5HRM5	gltX	fig|176279.9.peg.159	J	Glutamate--tRNA ligase	-1000.00	-1000.00	4.77
SERP0177	Q5HRL6	nusG	fig|176279.9.peg.168	K	Transcription termination/antitermination protein NusG	0.00	1000.00	1000.00
SERP0178	Q5HRL5	rplK	fig|176279.9.peg.169	J	50S ribosomal protein L11	3.43	2.77	1.34
SERP0179	Q5HRL4	rplA	fig|176279.9.peg.170	J	50S ribosomal protein L1	2.54	10.12	12.84
SERP0180	Q5HRL3	rplJ	fig|176279.9.peg.171	J	50S ribosomal protein L10	-1.40	2.38	2.07
SERP0181	Q5HRL2	rplL	fig|176279.9.peg.172	J	50S ribosomal protein L7/L12	1.81	2.02	1.16
SERP0183	Q5HRL0	rpoB	fig|176279.9.peg.174	K	DNA-directed RNA polymerase subunit beta	16.65	9.65	267.97
SERP0184	Q5HRK9	rpoC	fig|176279.9.peg.175	K	DNA-directed RNA polymerase subunit beta'	0.00	1000.00	1000.00
SERP0186	Q5HRK7	rpsL	fig|176279.9.peg.177	J	30S ribosomal protein S12	1000.00	0.00	1000.00
SERP0187	Q5HRK6	rpsG	fig|176279.9.peg.178	J	30S ribosomal protein S7	11.20	11.48	13.52
SERP0188	Q5HRK5	fusA	fig|176279.9.peg.179	J	Elongation factor G	3.29	6.15	8.75
SERP0189	Q5HRK4	tuf	fig|176279.9.peg.180	J	Elongation factor Tu	-6.12	-2.74	-1.25
SERP0199	Q5HRJ4	SERP0199	fig|176279.9.peg.190	S	Hydrolase, haloacid dehalogenase-like family	-5.41	2.36	1.88
SERP0200	Q5HRJ3	SERP0200	fig|176279.9.peg.191	C	Bac_luciferase domain-containing protein	0.00	1000.00	0.00
SERP0205	Q5HRI8	SERP0205	fig|176279.9.peg.196	S	Hydrolase, haloacid dehalogenase-like family	-1000.00	-3.43	5.77
SERP0206	Q5HRI7	azo1	fig|176279.9.peg.197	S	FMN-dependent NADPH-azoreductase	0.00	1000.00	1000.00
SERP0212	Q5HRI1	folE2	fig|176279.9.peg.203	S	GTP cyclohydrolase FolE2	0.00	0.00	1000.00
SERP0213	Q5HRI0	SERP0213	fig|176279.9.peg.204	S	Uncharacterized protein	-1000.00	1.25	5.23
SERP0217	Q5HRH6	SERP0217	fig|176279.9.peg.208	M	SIS domain protein	-132.95	1.43	1.93
SERP0226	Q5HRG7	pdxK	fig|176279.9.peg.217	H	Putative pyridoxine kinase	-34.66	-6.09	1.13
SERP0229	Q5HRG4	SERP0229	fig|176279.9.peg.220	S	Uncharacterized protein	14.37	1.69	7.62

SERP0236	Q5HRF7	pta	fig|176279.9.peg.226	C	Phosphate acetyltransferase	-4.02	-1.63	-1.50
SERP0244	Q5HRE9	SERP0244	fig|176279.9.peg.234	C	Oxidoreductase, aldo/keto reductase family	-5.81	-1.50	1.24
SERP0252	Q5HRE1	SERP0252	fig|176279.9.peg.241	S	Uncharacterized protein	0.00	0.00	1000.00
SERP0256	Q5HRD7	SERP0256	fig|176279.9.peg.245	S	Conserved domain protein	0.00	0.00	1000.00
SERP0257	Q5HRD6	adh	fig|176279.9.peg.246	C	Alcohol dehydrogenase	-21.31	-4.25	-2.32
SERP0262	Q5HRD1	argS	fig|176279.9.peg.250	J	Arginine--tRNA ligase	-1000.00	4.25	55.70
SERP0266	Q5HRC7	SERP0266	fig|176279.9.peg.253	P	Iron compound ABC transporter, iron compound-binding protein, putative	3.13	4.31	7.35
SERP0270	Q5HRC3	SERP0270	fig|176279.9.peg.257	-	Uncharacterized protein	1000.00	1000.00	1000.00
SERP0274	Q5HRB9	sarA	fig|176279.9.peg.261	K	Staphylococcal accessory regulator A	1000.00	0.00	1000.00
SERP0289	Q5HRA4	SERP0289	fig|176279.9.peg.274	P	Na+/H+ antiporter, putative	-1000.00	-1000.00	-1000.00
SERP0290	Q5HRA3	sitC	fig|176279.9.peg.275	P	ABC transporter, substrate-binding protein	1.25	-1.28	-2.55
SERP0311	Q5HR82	SERP0311	fig|176279.9.peg.295	GM	Uncharacterized protein	-1000.00	-1000.00	1.15
SERP0316	Q5HR77	SERP0316	fig|176279.9.peg.300	P	Uncharacterized protein	0.00	0.00	1000.00
SERP0318	Q5HR75	SERP0318	fig|176279.9.peg.302	M	LysM domain protein	1000.00	1000.00	1000.00
SERP0322	Q5HR71	SERP0322	fig|176279.9.peg.306	K	Probable transcriptional regulatory protein SERP0322	-6.52	-1.58	-1.54
SERP0324	Q5HR69	scdA	fig|176279.9.peg.308	D	Iron-sulfur cluster repair protein ScdA	-1.84	-2.93	-2.05
SERP0325	Q5HR68	SERP0325	fig|176279.9.peg.309	C	Flavohemoprotein, putative	-2.95	-1.39	-2.82
SERP0332	Q5HR61	SERP0332	fig|176279.9.peg.316	-	Lipoprotein, putative	0.00	1000.00	1000.00
SERP0333	Q5HR60	SERP0333	fig|176279.9.peg.317	-	Uncharacterized protein	-2.55	-5.98	6.83
SERP0337	Q5HR56	SERP0337	fig|176279.9.peg.321	-	Uncharacterized protein	0.00	1000.00	1000.00
SERP0342	Q5HR51	norR	fig|176279.9.peg.326	K	Transcriptional regulator, MarR family	1.06	3.78	5.59
SERP0354	Q5HR39	SERP0354	fig|176279.9.peg.336	-	Uncharacterized protein	0.00	1000.00	0.00
SERP0358	Q5HR35	fruK	fig|176279.9.peg.339	H	Tagatose-6-phosphate kinase	-3.32	-2.77	-1.99
SERP0359	Q5HR34	fruA	fig|176279.9.peg.340	G	PTS system, fructose-specific IIABC components	2.20	3.41	4.07
SERP0367	Q5HR26	SERP0367	fig|176279.9.peg.348	S	Lipoprotein, putative	2.16	1.73	1.76

SERP0372	Q5HR21	SERP0372	fig|176279.9.peg.353	H	6-carboxy-5,6,7,8-tetrahydropterin synthase	1.48	2.39	1.49
SERP0379	Q5HR16	ltaS	fig|176279.9.peg.361	M	Lipoteichoic acid synthase	1000.00	1000.00	1000.00
SERP0386	Q5HR09	SERP0386	fig|176279.9.peg.368	P	ABC transporter, permease protein	-1000.00	-1000.00	-1000.00
SERP0389	Q5HR06	SERP0389	fig|176279.9.peg.371	C	Aldehyde-alcohol dehydrogenase	-1000.00	-1000.00	-7.45
SERP0390	Q5HR05	SERP0390	fig|176279.9.peg.372	F	Putative lipid kinase SERP0390	-1000.00	-1000.00	-1000.00
SERP0402	Q5HQZ4	SERP0402	fig|176279.9.peg.382	P	Iron compound ABC transporter, ATP-binding protein	-1000.00	-1000.00	-1000.00
SERP0403	Q5HQZ3	SERP0403	fig|176279.9.peg.383	P	Transferrin receptor	1000.00	1000.00	1000.00
SERP0405	Q5HQZ1	murB	fig|176279.9.peg.385	M	UDP-N-acetylenolpyruvoylglucosamine reductase	0.00	0.00	1000.00
SERP0407	Q5HQY9	SERP0407	fig|176279.9.peg.387	-	Lipoprotein, putative	-1000.00	2.71	1.51
SERP0408	Q5HQY8	SERP0408	fig|176279.9.peg.388	O	Uncharacterized protein	-1.23	2.98	1.77
SERP0410	Q5HQY6	pepT	fig|176279.9.peg.390	E	Peptidase T	-1000.00	-1000.00	37.38
SERP0419	Q5HQX7	hpf	fig|176279.9.peg.399	J	Ribosome hibernation promotion factor	-1000.00	3.25	-1000.00
SERP0420	Q5HQX6	secA1	fig|176279.9.peg.400	U	Protein translocase subunit SecA 1	-1000.00	-2.93	-1000.00
SERP0421	Q5HQX5	prfB	fig|176279.9.peg.401	J	Peptide chain release factor 2	-1000.00	-1000.00	4.18
SERP0422	Q5HQX4	SERP0422	fig|176279.9.peg.402	M	LysM domain protein	0.00	1000.00	0.00
SERP0432	Q5HQW4	trxB	fig|176279.9.peg.411	C	Thioredoxin reductase	-14.37	-1.81	-1.03
SERP0436	Q5HQW0	clpP	fig|176279.9.peg.415	OU	ATP-dependent Clp protease proteolytic subunit	-3.67	-1.81	-2.11
SERP0439	Q5HQV7	SERP0439	fig|176279.9.peg.418	S	Uncharacterized protein	0.00	1000.00	1000.00
SERP0441	Q5HQV5	gapR	fig|176279.9.peg.420	K	Gap transcriptional regulator	0.00	0.00	1000.00
SERP0442	Q5HQV4	gapA1	fig|176279.9.peg.421	G	Glyceraldehyde-3-phosphate dehydrogenase 1	-7.83	-7.93	-1.92
SERP0443	Q5HQV3	pgk	fig|176279.9.peg.422	F	Phosphoglycerate kinase	-2.37	-1.60	-1.79
SERP0445	Q5HQV1	gpmI	fig|176279.9.peg.424	G	2,3-bisphosphoglycerate-independent phosphoglycerate mutase	-5.28	-1.44	1.04
SERP0449	Q5HQU7	est	fig|176279.9.peg.428	S	Carboxylesterase	0.00	0.00	1000.00
SERP0466	Q5HQT1	SERP0466	fig|176279.9.peg.445	K	Cold shock protein, CSD family	11.81	4.59	9.20
SERP0468	Q5HQS9	SERP0468	fig|176279.9.peg.447	-	Uncharacterized protein	-1000.00	1.90	1.04

SERP0477	Q5HQS0	SERP0477	fig|176279.9.peg.457	J	Acetyltransferase, GNAT family	0.00	0.00	1000.00
SERP0480	Q5HQR8	SERP0480	fig|176279.9.peg.460	O	Organic hydroperoxide resistance protein-like 1	1.56	-5.47	-3.27
SERP0484	Q5HQR4	SERP0484	fig|176279.9.peg.464	P	Uncharacterized protein	4.32	3.30	5.30
SERP0485	Q5HQR3	gcvH	fig|176279.9.peg.465	E	Glycine cleavage system H protein	1.82	1.20	-5.16
SERP0491	Q5HQQ7	SERP0491	fig|176279.9.peg.470	M	Lipoprotein	2.56	2.87	2.90
SERP0497	Q5HQQ1	sufD	fig|176279.9.peg.475	O	FeS assembly protein SufD	-1000.00	-26.93	1.65
SERP0498	Q5HQQ0	csd	fig|176279.9.peg.476	E	Probable cysteine desulfurase	-1000.00	-1000.00	-1000.00
SERP0499	Q5HQP9	SERP0499	fig|176279.9.peg.477	C	NifU domain protein	2.19	2.20	1.12
SERP0500	Q5HQP8	SERP0500	fig|176279.9.peg.478	O	UPF0051 protein SERP0500	0.00	0.00	1000.00
SERP0512	Q5HQN6	SERP0512	fig|176279.9.peg.490	S	Uncharacterized protein	0.00	0.00	1000.00
SERP0514	Q5HQN4	SERP0514	fig|176279.9.peg.492	S	Uncharacterized protein	2.99	2.00	2.50
SERP0515	Q5HQN3	nagD	fig|176279.9.peg.493	G	Acid sugar phosphatase	-1000.00	-1.04	5.28
SERP0518	Q5HQN0	dltA	fig|176279.9.peg.496	Q	D-alanine--D-alanyl carrier protein ligase	0.00	1000.00	0.00
SERP0520	Q5HQM8	dltC	fig|176279.9.peg.498	IQ	D-alanyl carrier protein	-1000.00	-106.80	-1.94
SERP0522	Q5HQM6	SERP0522	fig|176279.9.peg.500	O	NifU domain protein	-1000.00	1.49	-3.42
SERP0525	Q5HQM3	SERP0525	fig|176279.9.peg.503	S	UPF0349 protein SERP0525	1000.00	0.00	1000.00
SERP0526	Q5HQM2	SERP0526	fig|176279.9.peg.504	S	HesB domain protein	1000.00	1000.00	1000.00
SERP0527	Q5HQM1	SERP0527	fig|176279.9.peg.505	C	NADH dehydrogenase-like protein SERP0527	0.00	1000.00	1000.00
SERP0540	Q5HQK8	SERP0540	fig|176279.9.peg.518	O	Putative peptidyl-prolyl cis-trans isomerase	2.55	2.44	1.91
SERP0541	Q5HQK7	SERP0541	fig|176279.9.peg.519	J	General stress protein 13	0.00	1000.00	1000.00
SERP0542	Q5HQK6	SERP0542	fig|176279.9.peg.520	C	NADH-dependent flavin oxidoreductase, Oye family	-1000.00	-1000.00	-4.41
SERP0545	Q5HQK4	rocD	fig|176279.9.peg.525	E	Ornithine aminotransferase	0.00	0.00	1000.00
SERP0549	Q5HQK0	argG	fig|176279.9.peg.529	E	Argininosuccinate synthase	-1000.00	-1000.00	-1000.00
SERP0550	Q5HQJ9	pgi	fig|176279.9.peg.530	G	Glucose-6-phosphate isomerase	-1000.00	-50.03	1.29
SERP0553	Q5HQJ6	spsB	fig|176279.9.peg.533	U	Signal peptidase I	3.77	12.13	14.23
SERP0556	Q5HQJ3	SERP0556	fig|176279.9.peg.536	Q	Uncharacterized protein SERP0556	-2.13	-1.83	-1.27
SERP0562	Q5HQI7	SERP0562	fig|176279.9.peg.543	S	FeS_assembly_P domain-containing protein	0.00	0.00	1000.00

SERP0564	Q5HQI5	clpB	fig|176279.9.peg.545	O	Chaperone protein ClpB	-1000.00	-1.46	3.85
SERP0568	Q5HQI1	fabF	fig|176279.9.peg.549	I	3-oxoacyl-[acyl-carrier-protein] synthase 2	-16.63	-1.86	1.00
SERP0575	Q5HQH4	trpS	fig|176279.9.peg.556	J	Tryptophan--tRNA ligase	0.00	0.00	1000.00
SERP0576	Q5HQH3	spxA	fig|176279.9.peg.557	K	Regulatory protein Spx	-10.29	-1.18	3.74
SERP0578	Q5HQH1	mecA	fig|176279.9.peg.559	NOT	Adapter protein MecA	0.00	0.00	1000.00
SERP0580	Q5HQG9	pepF	fig|176279.9.peg.561	E	Oligoendopeptidase F	-1000.00	-1000.00	1.53
SERP0604	Q5HQE9	SERP0604	fig|176279.9.peg.585	J	Putative phosphoesterase SERP0604	-1000.00	-1000.00	1.24
SERP0611	Q5HQE2	SERP0611	fig|176279.9.peg.592	O	Serine protease HtrA-like	1.34	1.99	2.02
SERP0615	Q5HQD8	SERP0615	fig|176279.9.peg.596	S	Uncharacterized protein	9.56	1.55	3.20
SERP0629	Q5HQC6	SERP0629	fig|176279.9.peg.610	HQ	Isochorismate synthase family protein	-1000.00	-1000.00	-1000.00
SERP0632	Q5HQC3	menB	fig|176279.9.peg.613	H	1,4-dihydroxy-2-naphthoyl-CoA synthase	0.00	1000.00	1000.00
SERP0634	Q5HQC1	SERP0634	fig|176279.9.peg.615	G	Membrane protein, putative	-1000.00	-1000.00	-1000.00
SERP0636	Q5HQB9	atl	fig|176279.9.peg.617	M	Bifunctional autolysin	3.34	2.83	-1.40
SERP0640	Q5HQB5	SERP0640	fig|176279.9.peg.620	K	Transcriptional regulator, putative	1000.00	1000.00	1000.00
SERP0645	Q5HQB0	qoxB	fig|176279.9.peg.625	C	Probable quinol oxidase subunit 1	1000.00	0.00	1000.00
SERP0646	Q5HQA9	qoxA	fig|176279.9.peg.626	C	Probable quinol oxidase subunit 2	-1.01	2.95	5.85
SERP0651	Q5HQA4	purC	fig|176279.9.peg.631	F	Phosphoribosylaminoimidazole-succinocarboxamide synthase	1.76	4.61	-1.75
SERP0652	Q5HQA3	purS	fig|176279.9.peg.632	F	Phosphoribosylformylglycinamidine synthase subunit PurS	-1.29	-1.50	-10.02
SERP0653	Q5HQA2	purQ	fig|176279.9.peg.633	F	Phosphoribosylformylglycinamidine synthase subunit PurQ	-1000.00	-1000.00	-2.00
SERP0654	Q5HQA1	purL	fig|176279.9.peg.634	F	Phosphoribosylformylglycinamidine synthase subunit PurL	0.00	0.00	1000.00
SERP0656	Q5HQ99	purM	fig|176279.9.peg.636	F	Phosphoribosylformylglycinamidine cyclo-ligase	-1000.00	-1000.00	-1.81
SERP0657	Q5HQ98	purN	fig|176279.9.peg.637	F	Phosphoribosylglycinamide formyltransferase	-1000.00	-8.98	-5.48
SERP0665	Q5HQ90	SERP0665	fig|176279.9.peg.644	-	Uncharacterized protein	3.66	-5.96	2.79
SERP0667	Q5HQ88	SERP0667	fig|176279.9.peg.646	J	Uncharacterized protein	0.00	1000.00	0.00
SERP0668	Q5HQ87	SERP0668	fig|176279.9.peg.647	S	Uncharacterized protein	1000.00	0.00	1000.00

SERP0669	Q5HQ86	ptsH	fig|176279.9.peg.648	G	Phosphocarrier protein HPr	1.79	-2.91	-2.71
SERP0670	Q5HQ85	ptsI	fig|176279.9.peg.649	G	Phosphoenolpyruvate-protein phosphotransferase	-20.94	-1000.00	-4.77
SERP0674	Q5HQ81	SERP0674	fig|176279.9.peg.653	P	Potassium uptake protein TrkA	-1000.00	107.79	-1000.00
SERP0677	Q5HQ79	SERP0677	fig|176279.9.peg.655	S	UPF0356 protein SERP0677	25.47	16.00	6.53
SERP0678	Q5HQ78	def	fig|176279.9.peg.656	J	Peptide deformylase	-13.59	-2.41	1.03
SERP0679	Q5HQ77	SERP0679	fig|176279.9.peg.657	L	Uncharacterized protein	5.49	3.77	4.87
SERP0680	Q5HQ76	pdhA	fig|176279.9.peg.658	C	Pyruvate dehydrogenase E1 component subunit alpha	1.04	-1.94	2.62
SERP0681	Q5HQ75	pdhB	fig|176279.9.peg.659	C	Pyruvate dehydrogenase E1 component subunit beta	-49.97	-6.85	-1.09
SERP0683	Q5HQ73	pdhD	fig|176279.9.peg.661	C	Dihydrolipoyl dehydrogenase	-4.38	-3.76	3.50
SERP0684	Q5HQ72	SERP0684	fig|176279.9.peg.662	S	UPF0223 protein SERP0684	0.00	0.00	1000.00
SERP0690	Q5HQ66	SERP0690	fig|176279.9.peg.668	S	DUF4064 domain-containing protein	5.24	142.03	52.84
SERP0691	Q5HQ65	SERP0691	fig|176279.9.peg.669	S	DUF4064 domain-containing protein	-1000.00	1.24	-1.37
SERP0693	Q5HQ63	SERP0693	fig|176279.9.peg.671	S	UPF0637 protein SERP0693	1000.00	0.00	1000.00
SERP0694	Q5HQ62	SERP0694	fig|176279.9.peg.672	G	Inositol monophosphatase family protein	1000.00	0.00	0.00
SERP0696	Q5HQ60	SERP0696	fig|176279.9.peg.674	T	GTP-binding protein TypA	0.00	0.00	1000.00
SERP0700	Q5HQ57	SERP0700	fig|176279.9.peg.681	S	Uncharacterized protein	-1000.00	-1000.00	50.46
SERP0701	Q5HQ56	SERP0701	fig|176279.9.peg.682	S	UPF0358 protein SERP0701	0.00	0.00	1000.00
SERP0708	Q5HQ49	SERP0708	fig|176279.9.peg.688	S	Uncharacterized protein	0.00	1000.00	1000.00
SERP0717	Q5HQ40	SERP0717	fig|176279.9.peg.696	S	Uncharacterized protein	13.86	14.24	-1000.00
SERP0719	Q5HQ38	SERP0719	fig|176279.9.peg.698	S	Cell wall surface anchor family protein	0.00	1000.00	1000.00
SERP0721	Q5HQ36	pheS	fig|176279.9.peg.700	J	Phenylalanine--tRNA ligase alpha subunit	-1000.00	-1000.00	-13.26
SERP0724	Q5HQ33	SERP0724	fig|176279.9.peg.703	D	Cell division protein ZapA	-2.65	-1.12	-1.86
SERP0729	Q5HQ28	uvrC	fig|176279.9.peg.708	L	UvrABC system protein C	-1000.00	-1000.00	-1000.00
SERP0732	Q5HQ25	SERP0732	fig|176279.9.peg.711	C	Succinate dehydrogenase, iron-sulfur protein	-1000.00	41.43	80.17
SERP0734	Q5HQ23	SERP0734	fig|176279.9.peg.713	F	dITP/XTP pyrophosphatase	-1.79	1.61	3.03

SERP0738	Q5HQ19	SERP0738	fig|176279.9.peg.717	S	Phenol soluble modulin beta 1	1000.00	1000.00	0.00
SERP0743	Q5HQ14	mraZ	fig|176279.9.peg.722	K	Transcriptional regulator MraZ	0.00	0.00	1000.00
SERP0746	Q5HQ11	pbp1	fig|176279.9.peg.725	M	Penicillin-binding protein 1	1000.00	1000.00	1000.00
SERP0749	Q5HQ08	divIB	fig|176279.9.peg.728	D	Cell division protein DivIB	44.80	45.75	15.05
SERP0750	Q5HQ07	ftsA	fig|176279.9.peg.729	D	Cell division protein FtsA	-49.16	-28.29	-1.27
SERP0754	Q5HQ03	sepF	fig|176279.9.peg.733	D	Cell division protein SepF	-1.55	-3.67	-1.08
SERP0755	Q5HQ02	ylmG	fig|176279.9.peg.734	S	YlmG protein	0.00	0.00	1000.00
SERP0758	Q5HPZ9	ileS	fig|176279.9.peg.737	J	Isoleucine--tRNA ligase	0.00	0.00	1000.00
SERP0764	Q5HPZ3	pyrR	fig|176279.9.peg.742	F	Bifunctional protein PyrR	0.00	0.00	1000.00
SERP0767	Q5HPZ0	pyrC	fig|176279.9.peg.745	F	Dihydroorotase	0.00	1000.00	0.00
SERP0768	Q5HPY9	carA	fig|176279.9.peg.746	F	Carbamoyl-phosphate synthase small chain	0.00	1000.00	1000.00
SERP0769	Q5HPY8	carB	fig|176279.9.peg.747	F	Carbamoyl-phosphate synthase large chain	0.00	0.00	1000.00
SERP0771	Q5HPY6	pyrE	fig|176279.9.peg.749	F	Orotate phosphoribosyltransferase	-1000.00	-1.21	1.15
SERP0774	Q5HPY3	SERP0774	fig|176279.9.peg.752	S	3-dmu-9_3-mt domain-containing protein	0.00	0.00	1000.00
SERP0776	Q5HPY1	gmk	fig|176279.9.peg.754	F	Guanylate kinase	0.00	0.00	1000.00
SERP0781	Q5HPX6	SERP0781	fig|176279.9.peg.759	J	Peptide deformylase-like	0.00	0.00	1000.00
SERP0786	Q5HPX1	SERP0786	fig|176279.9.peg.764	KLT	Serine/threonine protein kinase, putative	0.00	1000.00	1000.00
SERP0788	Q5HPW9	rpe	fig|176279.9.peg.766	G	Ribulose-phosphate 3-epimerase	0.00	0.00	1000.00
SERP0792	Q5HPW5	SERP0792	fig|176279.9.peg.770	S	Uncharacterized protein SERP0792	-1000.00	3.24	13.45
SERP0795	Q5HPW2	plsX	fig|176279.9.peg.773	I	Phosphate acyltransferase	1.37	6.47	281.19
SERP0796	Q5HPW1	fabD	fig|176279.9.peg.774	I	Malonyl CoA-acyl carrier protein transacylase	-1000.00	-1.14	3.27
SERP0798	Q5HPV9	acpP	fig|176279.9.peg.776	IQ	Acyl carrier protein	-3.06	-2.18	-1.00
SERP0803	Q5HPV4	ffh	fig|176279.9.peg.781	U	Signal recognition particle protein	1000.00	1000.00	1000.00
SERP0804	Q5HPV3	rpsP	fig|176279.9.peg.782	J	30S ribosomal protein S16	11.89	13.39	21.72
SERP0805	Q5HPV2	rimM	fig|176279.9.peg.783	J	Ribosome maturation factor RimM	0.00	0.00	1000.00
SERP0813	Q5HPU5	sucC	fig|176279.9.peg.792	F	Succinate--CoA ligase [ADP-forming] subunit beta	-1000.00	-4.05	2.88

SERP0814	Q5HPU4	sucD	fig|176279.9.peg.793	C	Succinate--CoA ligase [ADP-forming] subunit alpha	-1000.00	-1.18	2.54
SERP0817	Q5HPU1	trmFO	fig|176279.9.peg.797	J	Methylenetetrahydrofolate--tRNA-(uracil-5-)-methyltransferase TrmFO	0.00	0.00	1000.00
SERP0818	Q5HPU0	xerC	fig|176279.9.peg.798	D	Tyrosine recombinase XerC	0.00	0.00	1000.00
SERP0820	Q5HPT8	hslU	fig|176279.9.peg.800	O	ATP-dependent protease ATPase subunit HslU	-1000.00	1.68	20.57
SERP0821	Q5HPT7	codY	fig|176279.9.peg.801	K	GTP-sensing transcriptional pleiotropic repressor CodY	-1000.00	-4.63	6.86
SERP0823	Q5HPT5	rpsB	fig|176279.9.peg.802	J	30S ribosomal protein S2	3.97	5.29	6.00
SERP0825	Q5HPT3	pyrH	fig|176279.9.peg.804	F	Uridylate kinase	-1000.00	-1000.00	-1000.00
SERP0826	Q5HPT2	frr	fig|176279.9.peg.805	J	Ribosome-recycling factor	2.31	-1.19	1.36
SERP0830	Q5HPS8	proS	fig|176279.9.peg.809	J	Proline--tRNA ligase	-1000.00	-1000.00	10.57
SERP0832	Q5HPS6	rimP	fig|176279.9.peg.811	S	Ribosome maturation factor RimP	-1000.00	-3.70	-15.26
SERP0833	Q5HPS5	nusA	fig|176279.9.peg.812	K	Transcription termination/antitermination protein NusA	-4.89	-1.25	4.23
SERP0834	Q5HPS4	SERP0834	fig|176279.9.peg.813	K	DUF448 domain-containing protein	0.00	1000.00	1000.00
SERP0836	Q5HPS2	infB	fig|176279.9.peg.815	J	Translation initiation factor IF-2	-1000.00	1.16	8.71
SERP0840	Q5HPR8	rpsO	fig|176279.9.peg.819	J	30S ribosomal protein S15	1000.00	1000.00	1000.00
SERP0841	Q5HPR7	pnp	fig|176279.9.peg.820	J	Polyribonucleotide nucleotidyltransferase	-336.52	-1.13	-1000.00
SERP0842	Q5HPR6	rnj2	fig|176279.9.peg.821	J	Ribonuclease J 2	0.00	0.00	1000.00
SERP0844	Q5HPR4	SERP0844	fig|176279.9.peg.823	K	Transcriptional regulator, putative	0.00	0.00	1000.00
SERP0848	Q5HPR0	SERP0848	fig|176279.9.peg.827	S	ACT domain protein	0.00	0.00	1000.00
SERP0849	Q5HPQ9	SERP0849	fig|176279.9.peg.828	S	Transcriptional regulator, Cro/CI family	0.00	0.00	1000.00
SERP0852	Q5HPQ6	recA	fig|176279.9.peg.831	L	Protein RecA	0.00	0.00	1000.00
SERP0853	Q5HPQ5	rny	fig|176279.9.peg.832	S	Ribonuclease Y	-1000.00	-2.25	10.59
SERP0854	Q5HPQ4	SERP0854	fig|176279.9.peg.833	-	Uncharacterized protein	0.00	0.00	1000.00
SERP0859	Q5HPP9	SERP0859	fig|176279.9.peg.838	S	Thiamine_BP domain-containing protein	1.63	-1.31	2.79
SERP0867	Q5HPP1	glpK	fig|176279.9.peg.846	F	Glycerol kinase	0.00	0.00	1000.00

SERP0868	Q5HPP0	glpD	fig|176279.9.peg.847	C	Aerobic glycerol-3-phosphate dehydrogenase	0.00	0.00	1000.00
SERP0871	Q5HPN7	hfq	fig|176279.9.peg.850	J	RNA-binding protein Hfq	1000.00	1000.00	1000.00
SERP0872	Q5HPN6	gpxA-1	fig|176279.9.peg.851	O	Glutathione peroxidase	-1.61	-1.88	-5.31
SERP0876	Q5HPN2	glnA	fig|176279.9.peg.855	E	Glutamine synthetase	-1000.00	-1000.00	1.41
SERP0879	Q5HPN0	SERP0879	fig|176279.9.peg.858	S	Uncharacterized protein	-1000.00	-1000.00	-1000.00
SERP0884	Q5HPM6	SERP0884	fig|176279.9.peg.864	-	Uncharacterized protein	2.42	2.71	4.16
SERP0896	Q5HPL5	SERP0896	fig|176279.9.peg.875	E	Aspartokinase	-1000.00	-1000.00	-1000.00
SERP0901	Q5HPL0	SERP0901	fig|176279.9.peg.880	-	Uncharacterized protein	-1000.00	1.76	-1000.00
SERP0903	Q5HPK8	katA	fig|176279.9.peg.882	P	Catalase	1000.00	0.00	0.00
SERP0907	Q5HPK4	SERP0907	fig|176279.9.peg.886	S	Uncharacterized protein	0.00	1000.00	0.00
SERP0911	Q5HPK0	SERP0911	fig|176279.9.peg.889	S	UPF0291 protein SERP0911	2.60	-2.52	1.03
SERP0912	Q5HPJ9	tkt	fig|176279.9.peg.890	G	Transketolase	-1000.00	-2.32	3.24
SERP0914	Q5HPJ7	SERP0914	fig|176279.9.peg.891	-	UPF0154 protein SERP0914	4.53	2.42	4.25
SERP0918	Q5HPJ3	sbcC	fig|176279.9.peg.895	L	Nuclease SbcCD subunit C	-1000.00	-1000.00	-1000.00
SERP0921	Q5HPJ0	acnA	fig|176279.9.peg.898	C	Aconitate hydratase A	-1000.00	-1.25	1.16
SERP0930	Q5HPI1	mprF	fig|176279.9.peg.907	S	Phosphatidylglycerol lysyltransferase	0.00	0.00	1000.00
SERP0934	Q5HPH8	SERP0934	fig|176279.9.peg.912	G	Probable tautomerase SERP0934	2.22	-1.64	-1.62
SERP0960	Q5HPF2	pstS	fig|176279.9.peg.938	P	Phosphate-binding protein PstS	-1.59	-3.03	-10.88
SERP0961	Q5HPF1	cvfB	fig|176279.9.peg.939	S	Conserved virulence factor B	1000.00	0.00	0.00
SERP0962	Q5HPF0	SERP0962	fig|176279.9.peg.940	S	ABC transporter, ATP-binding protein	0.00	0.00	1000.00
SERP0963	Q5HPE9	lysC	fig|176279.9.peg.941	E	Aspartokinase	-1000.00	-1000.00	-1000.00
SERP0964	Q5HPE8	asd	fig|176279.9.peg.942	E	Aspartate-semialdehyde dehydrogenase	0.00	0.00	1000.00
SERP0965	Q5HPE7	dapA	fig|176279.9.peg.943	E	4-hydroxy-tetrahydrodipicolinate synthase	0.00	0.00	1000.00
SERP0972	Q5HPE0	cspA	fig|176279.9.peg.949	K	Cold shock protein CspA	9.20	6.79	6.02
SERP0974	Q5HPD8	SERP0974	fig|176279.9.peg.951	C	Acylphosphatase	0.00	1000.00	1000.00
SERP0976	Q5HPD6	SERP0976	fig|176279.9.peg.953	P	TelA-like protein SERP0976	-2.27	-1.39	1.51
SERP0979	Q5HPD3	SERP0979	fig|176279.9.peg.956	S	Uncharacterized protein SERP0979	-1000.00	-1000.00	2.05
SERP0980	Q5HPD2	SERP0980	fig|176279.9.peg.957	-	Uncharacterized protein	-10.58	1.41	-1000.00

SERP0985	Q5HPC7	odhB	fig|176279.9.peg.962	C	Dihydrolipoyllysine-residue succinyltransferase component of 2-oxoglutarate dehydrogenase complex	-1000.00	-1.08	-2.47
SERP0996	Q5HPB7	SERP0996	fig|176279.9.peg.970	M	Probable CtpA-like serine protease	0.00	0.00	1000.00
SERP1000	Q5HPB3	msrA-2	fig|176279.9.peg.974	O	Peptide methionine sulfoxide reductase MsrA	2.13	1.04	-1.28
SERP1001	Q5HPB2	SERP1001	fig|176279.9.peg.975	S	DegV family protein	0.00	0.00	1000.00
SERP1006	Q5HPA7	SERP1006	fig|176279.9.peg.980	S	UPF0403 protein SERP1006	1.33	-2.56	-3.32
SERP1007	Q5HPA6	SERP1007	fig|176279.9.peg.981	C	Nfu_N domain-containing protein	6.01	-1000.00	-1000.00
SERP1011	Q5HPA2	ebh	fig|176279.9.peg.985	D	Extracellular matrix-binding protein ebh	-1000.00	7.04	15.03
SERP1014	Q5HP99	SERP1014	fig|176279.9.peg.988	GM	NAD(P)-bd_dom domain-containing protein	0.00	0.00	1000.00
SERP1016	Q5HP97	gpsB	fig|176279.9.peg.990	D	Cell cycle protein GpsB	-2.57	1.31	-1.02
SERP1017	Q5HP96	SERP1017	fig|176279.9.peg.991	S	UPF0398 protein SERP1017	0.00	0.00	1000.00
SERP1020	Q5HP93	pbp2	fig|176279.9.peg.994	M	Penicillin-binding protein 2	1.45	8.42	16.25
SERP1023	Q5HP90	SERP1023	fig|176279.9.peg.997	L	DNA replication protein DnaD, putative	0.00	1000.00	1000.00
SERP1024	Q5HP89	asnS	fig|176279.9.peg.998	J	Asparagine--tRNA ligase	-1000.00	-1000.00	-4.18
SERP1029	Q5HP84	SERP1029	fig|176279.9.peg.1003	S	MazG domain-containing protein	0.00	1000.00	1000.00
SERP1030	Q5HP83	SERP1030	fig|176279.9.peg.1004	S	Uncharacterized protein	1000.00	1000.00	1000.00
SERP1040	Q5HP73	SERP1040	fig|176279.9.peg.1014	-	Uncharacterized protein	-2.76	-2.77	1.38
SERP1041	Q5HP72	hup	fig|176279.9.peg.1015	L	DNA-binding protein HU	-8.48	-1.26	-1.71
SERP1045	Q5HP68	cmk	fig|176279.9.peg.1019	F	Cytidylate kinase	-1000.00	-9.51	1.58
SERP1048	Q5HP65	ebpS	fig|176279.9.peg.1022	M	Probable elastin-binding protein EbpS	1.06	-1.22	-3.93
SERP1051	Q5HP62	fer	fig|176279.9.peg.1025	C	Ferredoxin	15.12	16.84	12.95
SERP1055	Q5HP58	srrA	fig|176279.9.peg.1028	T	DNA-binding response regulator SrrA	-18.19	-2.68	3.32
SERP1057	Q5HP56	scpB	fig|176279.9.peg.1030	D	Segregation and condensation protein B	0.00	0.00	1000.00
SERP1060	Q5HP53	xerD	fig|176279.9.peg.1033	D	Tyrosine recombinase XerD	-1000.00	-1000.00	-1000.00
SERP1061	Q5HP52	SERP1061	fig|176279.9.peg.1034	P	Transcriptional regulator, Fur family	-4.41	2.08	3.34

SERP1064	Q5HP49	SERP1064	fig|176279.9.peg.1037	S	Oxidoreductase, short-chain dehydrogenase/reductase family	-1000.00	-1000.00	-1000.00
SERP1065	Q5HP48	proC	fig|176279.9.peg.1038	E	Pyrroline-5-carboxylate reductase	-1000.00	-1000.00	-1000.00
SERP1066	Q5HP47	rnz	fig|176279.9.peg.1039	J	Ribonuclease Z	-1000.00	-1000.00	-1000.00
SERP1071	Q5HP42	gnd	fig|176279.9.peg.1043	G	6-phosphogluconate dehydrogenase, decarboxylating	-1000.00	-1000.00	1.83
SERP1075	Q5HP38	SERP1075	fig|176279.9.peg.1046	S	UPF0403 protein SERP1075	2.25	1.09	-2.07
SERP1076	Q5HP37	SERP1076	fig|176279.9.peg.1047	C	Dihydrolipoamide acetyltransferase component of pyruvate dehydrogenase complex	0.00	0.00	1000.00
SERP1078	Q5HP35	SERP1078	fig|176279.9.peg.1049	C	2-oxoisovalerate dehydrogenase, E1 component, alpha subunit	-2.59	-1.87	-1.89
SERP1080	Q5HP33	recN	fig|176279.9.peg.1051	L	DNA repair protein RecN	-1000.00	-1000.00	6.71
SERP1081	Q5HP32	argR	fig|176279.9.peg.1052	K	Arginine repressor	-2.01	-3.56	2.47
SERP1082	Q5HP31	ispA	fig|176279.9.peg.1053	H	Geranyltranstransferase	0.00	0.00	1000.00
SERP1083	Q5HP30	xseB	fig|176279.9.peg.1054	L	Exodeoxyribonuclease 7 small subunit	6.66	-2.63	1.43
SERP1086	Q5HP27	SERP1086	fig|176279.9.peg.1057	S	Uncharacterized protein	-158.57	-1.07	1.11
SERP1088	Q5HP25	accB	fig|176279.9.peg.1059	I	Biotin carboxyl carrier protein of acetyl-CoA carboxylase	1000.00	1000.00	1000.00
SERP1090	Q5HP23	argB	fig|176279.9.peg.1061	F	Acetylglutamate kinase	-1000.00	-1000.00	-1000.00
SERP1095	Q5HP18	SERP1095	fig|176279.9.peg.1066	-	Lipoprotein, putative	0.00	1000.00	1000.00
SERP1097	Q5HP16	lipM	fig|176279.9.peg.1068	H	Octanoyltransferase LipM	-1000.00	25.33	26.18
SERP1098	Q5HP15	SERP1098	fig|176279.9.peg.1069	P	Rhodanese-like domain protein	0.00	0.00	1000.00
SERP1100	Q5HP14	gcvPB	fig|176279.9.peg.1071	E	Probable glycine dehydrogenase (decarboxylating) subunit 2	0.00	1000.00	1000.00
SERP1111	Q5HP03	SERP1111	fig|176279.9.peg.1082	S	Thiamine_BP domain-containing protein	2.11	1.07	-1.30
SERP1112	Q5HP02	glk	fig|176279.9.peg.1083	G	Glucokinase	-1000.00	-1000.00	17.45
SERP1116	Q5HNZ8	rpmG1	fig|176279.9.peg.1087	J	50S ribosomal protein L33 1	0.00	1000.00	1000.00
SERP1117	Q5HNZ7	pbp3	fig|176279.9.peg.1088	M	Penicillin-binding protein 3	0.00	1000.00	1000.00
SERP1119	Q5HNZ5	sodA	fig|176279.9.peg.1089	C	Superoxide dismutase [Mn/Fe]	-1.13	1.04	-2.88
SERP1120	Q5HNZ4	fur	fig|176279.9.peg.1090	P	Ferric uptake regulation protein	-2.76	-2.16	-1.05

SERP1123	Q5HNZ1	nfo	fig|176279.9.peg.1093	L	Probable endonuclease 4	-1.77	2.42	3.16
SERP1124	Q5HNZ0	cshB	fig|176279.9.peg.1094	JKL	DEAD-box ATP-dependent RNA helicase CshB	0.00	1000.00	1000.00
SERP1127	Q5HNY7	sigA	fig|176279.9.peg.1097	K	RNA polymerase sigma factor SigA	3.91	4.99	27.12
SERP1132	Q5HNY2	glyQS	fig|176279.9.peg.1101	J	Glycine--tRNA ligase	-1000.00	2.70	9.91
SERP1137	Q5HNX7	ybeY	fig|176279.9.peg.1106	J	Endoribonuclease YbeY	2.13	-1.26	1.95
SERP1139	Q5HNX5	SERP1139	fig|176279.9.peg.1108	-	Uncharacterized protein	3.12	8.24	-2.03
SERP1140	Q5HNX4	SERP1140	fig|176279.9.peg.1109	S	UPF0365 protein SERP1140	2.09	4.12	6.79
SERP1147	Q5HNW7	dnaJ	fig|176279.9.peg.1116	O	Chaperone protein DnaJ	1.12	7.95	4.27
SERP1151	Q5HNW3	SERP1151	fig|176279.9.peg.1120	H	Heme chaperone HemW	-1000.00	-1000.00	-1000.00
SERP1153	Q5HNW1	rpsT	fig|176279.9.peg.1122	J	30S ribosomal protein S20	1.56	-1.17	3.74
SERP1159	Q5HNV5	rsfS	fig|176279.9.peg.1128	J	Ribosomal silencing factor RsfS	0.00	1000.00	1000.00
SERP1162	Q5HNV2	SERP1162	fig|176279.9.peg.1131	J	CRM domain-containing protein	2.22	4.26	9.12
SERP1169	Q5HNU5	SERP1169	fig|176279.9.peg.1138	I	Acetyl-CoA carboxylase, biotin carboxylase, putative	0.00	0.00	1000.00
SERP1170	Q5HNU4	SERP1170	fig|176279.9.peg.1139	I	Acetyl-CoA carboxylase, biotin carboxyl carrier protein, putative	-1000.00	-1000.00	-1000.00
SERP1175	Q5HNT9	udk	fig|176279.9.peg.1144	F	Uridine kinase	0.00	0.00	1000.00
SERP1176	Q5HNT8	SERP1176	fig|176279.9.peg.1145	O	Peptidase, U32 family	-1000.00	-1000.00	-29.96
SERP1179	Q5HNT5	SERP1179	fig|176279.9.peg.1148	S	UPF0473 protein SERP1179	4.83	3.16	1.24
SERP1181	Q5HNT3	SERP1181	fig|176279.9.peg.1150	S	UPF0297 protein SERP1181	-1.09	-6.24	-1.44
SERP1184	Q5HNT0	SERP1184	fig|176279.9.peg.1153	S	TPR domain protein	0.00	0.00	1000.00
SERP1187	Q5HNS7	SERP1187	fig|176279.9.peg.1156	C	Bacterial luciferase family protein	0.00	1000.00	1000.00
SERP1188	Q5HNS6	SERP1188	fig|176279.9.peg.1157	-	Uncharacterized protein	4.87	3.56	3.73
SERP1193	Q5HNS1	hisS	fig|176279.9.peg.1162	J	Histidine--tRNA ligase	-1000.00	-1000.00	-1000.00
SERP1194	Q5HNS0	lytH	fig|176279.9.peg.1163	M	Probable cell wall amidase LytH	0.00	0.00	1000.00
SERP1201	Q5HNR4	secD	fig|176279.9.peg.1170	U	Multifunctional fusion protein	0.00	1000.00	1000.00
SERP1202	Q5HNR3	yajC	fig|176279.9.peg.1171	U	Preprotein translocase, YajC subunit	-1000.00	1.50	2.70
SERP1208	Q5HNQ7	obg	fig|176279.9.peg.1177	S	GTPase Obg	0.00	0.00	1000.00
SERP1209	Q5HNQ6	rpmA	fig|176279.9.peg.1178	J	50S ribosomal protein L27	5.89	4.72	1.09
SERP1211	Q5HNQ4	rplU	fig|176279.9.peg.1180	J	50S ribosomal protein L21	10.30	16.59	19.34
SERP1213	Q5HNQ2	mreC	fig|176279.9.peg.1182	M	Cell shape-determining protein MreC	0.00	1000.00	1000.00

SERP1214	Q5HNQ1	SERP1214	fig|176279.9.peg.1183	-	Uncharacterized protein	5.31	3.26	1.41
SERP1220	Q5HNP6	ermA-1	fig|176279.9.peg.1187	J	Tn554, rRNA adenine N-6-methyltransferase	0.00	0.00	1000.00
SERP1231	Q5HNN6	hemL1	fig|176279.9.peg.1198	H	Glutamate-1-semialdehyde 2,1-aminomutase 1	-1000.00	-57.67	2.50
SERP1238	Q5HNM9	clpX	fig|176279.9.peg.1205	O	ATP-dependent Clp protease ATP-binding subunit ClpX	-2.08	1.22	2.98
SERP1241	Q5HNM6	SERP1241	fig|176279.9.peg.1208	S	MutT/nudix family protein	-1000.00	-30.35	-1.83
SERP1242	Q5HNM5	rplT	fig|176279.9.peg.1209	J	50S ribosomal protein L20	1000.00	1000.00	1000.00
SERP1244	Q5HNM3	infC	fig|176279.9.peg.1211	J	Translation initiation factor IF-3	0.00	1000.00	1000.00
SERP1246	Q5HNM1	thrS	fig|176279.9.peg.1213	J	Threonine--tRNA ligase	0.00	0.00	1000.00
SERP1249	Q5HNL8	nrdR	fig|176279.9.peg.1216	K	Transcriptional repressor NrdR	0.00	1000.00	1000.00
SERP1252	Q5HNL6	coaE	fig|176279.9.peg.1220	F	Dephospho-CoA kinase	-2.36	-1.67	1.83
SERP1255	Q5HNL3	phoR	fig|176279.9.peg.1223	T	Sensory box histidine kinase PhoR	-1000.00	-1000.00	-1000.00
SERP1257	Q5HNL1	icd	fig|176279.9.peg.1225	C	Isocitrate dehydrogenase [NADP]	-5.93	1.27	2.92
SERP1258	Q5HNL0	gltA	fig|176279.9.peg.1226	C	Citrate synthase	0.00	0.00	1000.00
SERP1262	Q5HNK6	pfkA	fig|176279.9.peg.1230	F	ATP-dependent 6-phosphofructokinase	-4.48	-2.45	1.65
SERP1263	Q5HNK5	accA	fig|176279.9.peg.1231	I	Acetyl-coenzyme A carboxylase carboxyl transferase subunit alpha	0.00	0.00	1000.00
SERP1265	Q5HNK3	SERP1265	fig|176279.9.peg.1233	C	NADP-dependent malic enzyme, putative	0.00	1000.00	1000.00
SERP1266	Q5HNK2	dnaE	fig|176279.9.peg.1234	L	DNA polymerase III subunit alpha	0.00	0.00	1000.00
SERP1269	Q5HNJ9	SERP1269	fig|176279.9.peg.1237	T	Universal stress protein family	24.53	45.11	-1000.00
SERP1270	Q5HNJ8	SERP1270	fig|176279.9.peg.1238	S	UPF0173 metal-dependent hydrolase SERP1270	1.35	-1000.00	-2.62
SERP1271	Q5HNJ7	SERP1271	fig|176279.9.peg.1239	E	Uncharacterized peptidase SERP1271	0.00	0.00	1000.00
SERP1272	Q5HNJ6	ald	fig|176279.9.peg.1240	E	Alanine dehydrogenase	-2.44	-12.47	2.65
SERP1273	Q5HNJ5	SERP1273	fig|176279.9.peg.1241	T	Putative universal stress protein SERP1273	2.21	2.04	1.97
SERP1275	Q5HNJ4	ackA	fig|176279.9.peg.1243	F	Acetate kinase	0.00	1000.00	1000.00
SERP1276	Q5HNJ3	SERP1276	fig|176279.9.peg.1244	L	N6_Mtase domain-containing protein	0.00	0.00	1000.00

SERP1277	Q5HNJ2	tpx	fig|176279.9.peg.1245	O	Thiol peroxidase	-1.62	-2.02	-1.27
SERP1281	Q5HNI8	ezrA	fig|176279.9.peg.1249	D	Septation ring formation regulator EzrA	-2.27	1.35	2.26
SERP1282	Q5HNI7	SERP1282	fig|176279.9.peg.1250	T	GAF domain protein	-4.14	-2.16	-1.37
SERP1284	Q5HNI5	rpsD	fig|176279.9.peg.1252	J	30S ribosomal protein S4	-1000.00	33.17	295.23
SERP1290	Q5HNH9	SERP1290	fig|176279.9.peg.1258	G	PTS system, IIBC components	-1000.00	-1000.00	-1000.00
SERP1291	Q5HNH8	SERP1291	fig|176279.9.peg.1259	I	1-acyl-sn-glycerol-3-phosphate acyltransferase, putative	-1000.00	-1000.00	28.79
SERP1292	Q5HNH7	SERP1292	fig|176279.9.peg.1260	O	Serine protease HtrA, putative	-2.89	-1.39	2.34
SERP1296	Q5HNH3	ccpA	fig|176279.9.peg.1264	K	Catabolite control protein A	-1.02	5.43	40.64
SERP1297	Q5HNH2	SERP1297	fig|176279.9.peg.1265	E	Chorismate mutase/phospho-2-dehydro-3-deoxyheptonate aldolase	-1000.00	2.84	12.89
SERP1298	Q5HNH1	SERP1298	fig|176279.9.peg.1266	M	Uncharacterized protein	1.01	-1.26	2.07
SERP1301	Q5HNG8	SERP1301	fig|176279.9.peg.1269	D	FtsK/SpoIIIE family protein	-1000.00	23.95	6.40
SERP1306	Q5HNG3	SERP1306	fig|176279.9.peg.1274	S	PepSY domain-containing protein	0.00	1000.00	1000.00
SERP1309	Q5HNG0	dat	fig|176279.9.peg.1277	E	D-alanine aminotransferase	-13.82	-1.56	-2.50
SERP1310	Q5HNF9	SERP1310	fig|176279.9.peg.1278	E	Putative dipeptidase SERP1310	4.47	57.25	77.41
SERP1316	Q5HNF3	SERP1316	fig|176279.9.peg.1283	D	Cell wall surface anchor family protein	0.00	1000.00	1000.00
SERP1322	Q5HNE7	rot	fig|176279.9.peg.1289	K	HTH-type transcriptional regulator rot	-1000.00	-1.66	-1.43
SERP1325	Q5HNE4	ribH	fig|176279.9.peg.1292	H	6,7-dimethyl-8-ribityllumazine synthase	-6.04	-1000.00	-1000.00
SERP1330	Q5HND9	SERP1330	fig|176279.9.peg.1297	G	N-acetylmuramoyl-L-alanine amidase, family 4	21.04	86.37	126.30
SERP1336	Q5HND3	tal	fig|176279.9.peg.1303	H	Transaldolase	-1000.00	1.63	8.49
SERP1350	Q5HNC0	SERP1350	fig|176279.9.peg.1317	S	Oxidoreductase, aldo/keto reductase family	-1000.00	-1000.00	11.28
SERP1351	Q5HNB9	SERP1351	fig|176279.9.peg.1318	S	Uncharacterized protein	-4.92	-1.06	3.36
SERP1352	Q5HNB8	metK	fig|176279.9.peg.1319	H	S-adenosylmethionine synthase	0.00	1000.00	1000.00
SERP1367	Q5HNA5	hemH	fig|176279.9.peg.1335	H	Ferrochelatase	-1000.00	-1000.00	1.44
SERP1373	Q5HN99	SERP1373	fig|176279.9.peg.1341	FG	HIT family protein	1.13	-2.67	-1.03
SERP1374	Q5HN98	SERP1374	fig|176279.9.peg.1342	S	Uncharacterized protein	1.09	-1.75	3.18
SERP1376	Q5HN96	prsA	fig|176279.9.peg.1344	M	Foldase protein PrsA	3.76	1.31	-1.03

SERP1379	Q5HN93	SERP1379	fig|176279.9.peg.1347	D	AAA_27 domain-containing protein	0.00	0.00	1000.00
SERP1381	Q5HN91	SERP1381	fig|176279.9.peg.1349	S	UPF0342 protein SERP1381	-1.73	-2.44	-1.24
SERP1387	Q5HN85	fumC	fig|176279.9.peg.1355	C	Fumarate hydratase class II	-1000.00	19.99	5.65
SERP1390	Q5HN82	SERP1390	fig|176279.9.peg.1358	G	Glucosamine-6-phosphate isomerase family protein	-31.01	-4.77	-1.56
SERP1397	Q5HN75	gseA	fig|176279.9.peg.1366	M	Glutamyl endopeptidase	6.76	6.35	-1.20
SERP1398	Q5HN74	perR	fig|176279.9.peg.1367	K	Peroxide-responsive repressor PerR	-1000.00	-8.32	1.17
SERP1414	Q5HN58	SERP1414	fig|176279.9.peg.1382	-	Uncharacterized protein	0.00	1000.00	1000.00
SERP1417	Q5HN55	SERP1417	fig|176279.9.peg.1385	E	Aminopeptidase family protein	0.00	0.00	1000.00
SERP1418	Q5HN54	SERP1418	fig|176279.9.peg.1386	S	UPF0435 protein SERP1418	0.00	0.00	1000.00
SERP1419	Q5HN53	ptpA	fig|176279.9.peg.1387	T	Low molecular weight protein-tyrosine-phosphatase PtpA	-1000.00	-10.05	1.27
SERP1420	Q5HN52	SERP1420	fig|176279.9.peg.1388	S	Uncharacterized protein	3.57	2.07	-16.58
SERP1426	Q5HN46	map	fig|176279.9.peg.1394	E	Methionine aminopeptidase	-1.36	2.00	-2.47
SERP1431	Q5HN41	ftnA	fig|176279.9.peg.1399	P	Bacterial non-heme ferritin	-1000.00	-4.46	-2.42
SERP1435	Q5HN37	SERP1435	fig|176279.9.peg.1403	J	Uncharacterized RNA methyltransferase SERP1435	-1000.00	-1000.00	-1000.00
SERP1437	Q5HN35	gatB	fig|176279.9.peg.1405	J	Aspartyl/glutamyl-tRNA(Asn/Gln) amidotransferase subunit B	1.16	2.18	4.53
SERP1438	Q5HN34	gatA	fig|176279.9.peg.1406	J	Glutamyl-tRNA(Gln) amidotransferase subunit A	3.56	4.41	14.25
SERP1439	Q5HN33	gatC	fig|176279.9.peg.1407	J	Aspartyl/glutamyl-tRNA(Asn/Gln) amidotransferase subunit C	-9.71	-3.94	1.82
SERP1441	Q5HN31	camS	fig|176279.9.peg.1409	S	Staphylococcus epidermidis sex pheromone	2.92	3.69	9.20
SERP1449	Q5HN23	nadE	fig|176279.9.peg.1417	F	NH(3)-dependent NAD(+) synthetase	-1000.00	5.53	52.97
SERP1450	Q5HN22	SERP1450	fig|176279.9.peg.1418	H	Nicotinate phosphoribosyltransferase	0.00	0.00	1000.00
SERP1455	Q5HN17	ppaC	fig|176279.9.peg.1424	C	Probable manganese-dependent inorganic pyrophosphatase	-9.04	-2.65	-1.03
SERP1465	Q5HN08	SERP1465	fig|176279.9.peg.1434	L	Tn554-related, transposase B	0.00	0.00	1000.00
SERP1469	Q5HN05	SERP1469	fig|176279.9.peg.1438	S	Uncharacterized protein	0.00	0.00	1000.00

SERP1478	Q5HMZ7	SERP1478	fig|176279.9.peg.1447	K	Transcriptional regulator, GntR family	0.00	0.00	1000.00
SERP1483	Q5HMZ2	SERP1483	fig|176279.9.peg.1452	-	Cell wall surface anchor family protein	0.00	1000.00	1000.00
SERP1485	Q5HMZ0	groS	fig|176279.9.peg.1454	O	10 kDa chaperonin	-1.16	-5.06	-4.06
SERP1487	Q5HMY8	sdrH	fig|176279.9.peg.1456	DZ	SdrH protein	1000.00	1000.00	0.00
SERP1499	Q5HMX6	SERP1499	fig|176279.9.peg.1466	O	UPF0033 domain-containing protein	0.00	1000.00	1000.00
SERP1509	Q5HMW7	SERP1509	fig|176279.9.peg.1476	H	Uncharacterized protein	-1000.00	-1000.00	-1000.00
SERP1515	Q5HMW3	nrdI-2	fig|176279.9.peg.1482	F	NrdI protein	0.00	0.00	1000.00
SERP1517	Q5HMW1	SERP1517	fig|176279.9.peg.1484	-	Uncharacterized protein	0.00	0.00	1000.00
SERP1518	Q5HMW0	SERP1518	fig|176279.9.peg.1485	S	Uncharacterized protein	0.00	1000.00	0.00
SERP1542	Q5HMT6	SERP1542	fig|176279.9.peg.1508	-	Uncharacterized protein	7.22	3.79	1.04
SERP1543	Q5HMT5	SERP1543	fig|176279.9.peg.1509	-	Uncharacterized protein	2.74	-1.00	1.18
SERP1544	Q5HMT4	SERP1544	fig|176279.9.peg.1510	-	Lipoprotein, putative	0.00	1000.00	1000.00
SERP1550	Q5HMS8	SERP1550	fig|176279.9.peg.1516	-	Uncharacterized protein	1000.00	0.00	1000.00
SERP1580	Q5HMP8	SERP1580	fig|176279.9.peg.1545	S	Uncharacterized protein	0.00	0.00	1000.00
SERP1581	Q5HMP7	folA-2	fig|176279.9.peg.1546	H	Dihydrofolate reductase	0.00	0.00	1000.00
SERP1585	Q5HMP3	aacA-aphD	fig|176279.9.peg.1550	F	Bifunctional AAC/APH	0.00	0.00	1000.00
SERP1597	Q5HMN1	SERP1597	fig|176279.9.peg.1562	-	Uncharacterized protein	21.24	3.64	5.99
SERP1600	Q5HMM8	SERP1600	fig|176279.9.peg.1565	S	Uncharacterized protein	0.00	0.00	1000.00
SERP1605	Q5HMM3	SERP1605	fig|176279.9.peg.1569	-	Conserved domain protein	-1000.00	-1000.00	-1000.00
SERP1621	Q5HMK7	SERP1621	fig|176279.9.peg.1584	L	DNA-binding protein HU, putative	5.43	2.74	2.44
SERP1628	Q5HMK0	SERP1628	fig|176279.9.peg.1591	-	Uncharacterized protein	0.00	1000.00	0.00
SERP1629	Q5HMJ9	SERP1629	fig|176279.9.peg.1592	-	Uncharacterized protein	14.01	3.74	8.48
SERP1636	Q5HMJ2	SERP1636	fig|176279.9.peg.1599	-	Uncharacterized protein	12.60	5.15	1.64
SERP1642	Q5HMI6	SERP1642	fig|176279.9.peg.1605	S	Uncharacterized protein	0.00	0.00	1000.00
SERP1643	Q5HMI5	SERP1643	fig|176279.9.peg.1606	-	Uncharacterized protein	0.00	1000.00	0.00
SERP1677	Q5HMF1	rpoF	fig|176279.9.peg.1637	K	RNA polymerase sigma factor	5.35	50.29	105.90
SERP1678	Q5HMF0	rsbW	fig|176279.9.peg.1638	T	Serine-protein kinase RsbW	0.00	0.00	1000.00
SERP1679	Q5HME9	rsbV	fig|176279.9.peg.1639	T	Anti-sigma-B factor antagonist	2.04	1.84	3.73
SERP1688	Q5HME0	cshA	fig|176279.9.peg.1648	JKL	DEAD-box ATP-dependent RNA helicase CshA	0.00	1000.00	1000.00
SERP1690	Q5HMD8	ddl	fig|176279.9.peg.1650	F	D-alanine--D-alanine ligase	-1000.00	-1000.00	-1000.00

SERP1693	Q5HMD5	SERP1693	fig|176279.9.peg.1653	-	HMA domain-containing protein	-1.40	4.82	-1000.00
SERP1697	Q5HMD1	yidC2	fig|176279.9.peg.1657	U	Membrane protein insertase YidC 2	0.00	1000.00	1000.00
SERP1708	Q5HMC0	atpC	fig|176279.9.peg.1668	C	ATP synthase epsilon chain	0.00	0.00	1000.00
SERP1709	Q5HMB9	atpD	fig|176279.9.peg.1669	C	ATP synthase subunit beta	-11.30	-1.32	1.93
SERP1711	Q5HMB7	atpA	fig|176279.9.peg.1671	C	ATP synthase subunit alpha	5.44	9.02	18.54
SERP1712	Q5HMB6	atpH	fig|176279.9.peg.1672	C	ATP synthase subunit delta	2.75	3.61	4.80
SERP1718	Q5HMB1	upp	fig|176279.9.peg.1678	F	Uracil phosphoribosyltransferase	4.11	-1.17	13.59
SERP1720	Q5HMA9	SERP1720	fig|176279.9.peg.1680	S	UPF0340 protein SERP1720	0.00	1000.00	0.00
SERP1721	Q5HMA8	ptpB	fig|176279.9.peg.1681	T	Low molecular weight protein-tyrosine-phosphatase PtpB	0.00	0.00	1000.00
SERP1725	Q5HMA4	prfA	fig|176279.9.peg.1684	J	Peptide chain release factor 1	1.60	2.08	2.87
SERP1726	Q5HMA3	tdk	fig|176279.9.peg.1685	F	Thymidine kinase	0.00	0.00	1000.00
SERP1728	Q5HMA1	rho	fig|176279.9.peg.1687	K	Transcription termination factor Rho	0.00	0.00	1000.00
SERP1729	Q5HMA0	SERP1729	fig|176279.9.peg.1688	C	Putative aldehyde dehydrogenase SERP1729	-1.33	-1.93	2.11
SERP1735	Q5HM94	rpoE	fig|176279.9.peg.1694	K	Probable DNA-directed RNA polymerase subunit delta	137.81	143.50	22.87
SERP1738	Q5HM91	SERP1738	fig|176279.9.peg.1697	S	Uncharacterized protein	0.00	0.00	1000.00
SERP1741	Q5HM88	luxS	fig|176279.9.peg.1700	H	S-ribosylhomocysteine lyase	1.29	-2.85	-1.60
SERP1743	Q5HM86	deoB	fig|176279.9.peg.1702	G	Phosphopentomutase	-19.11	-4.55	-2.66
SERP1744	Q5HM85	pdp	fig|176279.9.peg.1703	F	Pyrimidine-nucleoside phosphorylase	-6.23	-1.87	-1.33
SERP1745	Q5HM84	deoC	fig|176279.9.peg.1704	F	Deoxyribose-phosphate aldolase	-1.70	-2.44	1.46
SERP1748	Q5HM81	SERP1748	fig|176279.9.peg.1706	P	Dps family protein	-1.05	-3.48	-11.92
SERP1754	Q5HM75	SERP1754	fig|176279.9.peg.1711	GM	NAD(P)-bd_dom domain-containing protein	-1000.00	1.15	22.66
SERP1758	Q5HM71	SERP1758	fig|176279.9.peg.1715	-	Lytic regulatory protein, putative	-1000.00	3.68	6.71
SERP1759	Q5HM70	SERP1759	fig|176279.9.peg.1716	S	Cof-like hydrolase, HAD-superfamily, subfamily IIB	-2.51	-1.31	-1.10
SERP1760	Q5HM69	glmS	fig|176279.9.peg.1717	M	Glutamine--fructose-6-phosphate aminotransferase [isomerizing]	0.00	0.00	1000.00
SERP1762	Q5HM67	glmM	fig|176279.9.peg.1718	G	Phosphoglucosamine mutase	-9.29	-1.66	7.39
SERP1763	Q5HM66	SERP1763	fig|176279.9.peg.1719	S	Uncharacterized protein	2.91	9.17	8.66

SERP1772	Q5HM57	SERP1772	fig|176279.9.peg.1728	-	UPF0457 protein SERP1772	-1000.00	-75.61	-35.95
SERP1777	Q5HM52	SERP1777	fig|176279.9.peg.1733	P	Iron compound ABC transporter, iron compound-binding protein	22.55	19.37	10.98
SERP1779	Q5HM50	SERP1779	fig|176279.9.peg.1735	Q	Uncharacterized protein	1000.00	0.00	0.00
SERP1783	Q5HM46	SERP1783	fig|176279.9.peg.1739	S	Uncharacterized protein	0.00	1000.00	1000.00
SERP1784	Q5HM45	SERP1784	fig|176279.9.peg.1740	S	Uncharacterized protein	4.25	7.34	19.00
SERP1786	Q5HM43	SERP1786	fig|176279.9.peg.1742	S	Alcohol dehydrogenase, zinc-containing	-1000.00	1.10	4.92
SERP1791	Q5HM39	lacF	fig|176279.9.peg.1748	G	PTS system lactose-specific EIIA component	-1.73	-1000.00	-1.41
SERP1798	Q5HM32	rpsI	fig|176279.9.peg.1755	J	30S ribosomal protein S9	5.07	12.83	17.64
SERP1799	Q5HM31	rplM	fig|176279.9.peg.1756	J	50S ribosomal protein L13	3.05	6.68	5.74
SERP1804	Q5HM26	rplQ	fig|176279.9.peg.1761	J	50S ribosomal protein L17	8.32	8.99	5.65
SERP1805	Q5HM25	rpoA	fig|176279.9.peg.1762	K	DNA-directed RNA polymerase subunit alpha	1.00	2.04	6.58
SERP1806	Q5HM24	rpsK	fig|176279.9.peg.1763	J	30S ribosomal protein S11	49.19	41.70	202.55
SERP1807	Q5HM23	rpsM	fig|176279.9.peg.1764	J	30S ribosomal protein S13	1000.00	1000.00	1000.00
SERP1809	Q5HM21	infA	fig|176279.9.peg.1766	J	Translation initiation factor IF-1	2.91	3.65	4.95
SERP1810	Q5HM20	adk	fig|176279.9.peg.1767	F	Adenylate kinase	2.04	2.50	2.89
SERP1812	Q5HM18	rplO	fig|176279.9.peg.1769	J	50S ribosomal protein L15	9.94	14.43	15.32
SERP1813	Q5HM17	rpmD	fig|176279.9.peg.1770	J	50S ribosomal protein L30	2.61	4.52	3.31
SERP1814	Q5HM16	rpsE	fig|176279.9.peg.1771	J	30S ribosomal protein S5	-3.48	6.69	11.59
SERP1815	Q5HM15	rplR	fig|176279.9.peg.1772	J	50S ribosomal protein L18	7.10	12.85	8.90
SERP1816	Q5HM14	rplF	fig|176279.9.peg.1773	J	50S ribosomal protein L6	26.76	36.00	49.69
SERP1817	Q5HM13	rpsH	fig|176279.9.peg.1774	J	30S ribosomal protein S8	3.32	6.06	11.55
SERP1819	Q5HM11	rplE	fig|176279.9.peg.1776	J	50S ribosomal protein L5	2.03	9.30	11.50
SERP1820	Q5HM10	rplX	fig|176279.9.peg.1777	J	50S ribosomal protein L24	2.71	2.98	2.39
SERP1821	Q5HM09	rplN	fig|176279.9.peg.1778	J	50S ribosomal protein L14	-1.70	-1000.00	33.34
SERP1823	Q5HM07	rpmC	fig|176279.9.peg.1780	J	50S ribosomal protein L29	4.32	6.88	9.17
SERP1824	Q5HM06	rplP	fig|176279.9.peg.1781	J	50S ribosomal protein L16	-1000.00	-1000.00	72.77
SERP1825	Q5HM05	rpsC	fig|176279.9.peg.1782	J	30S ribosomal protein S3	0.00	1000.00	1000.00
SERP1826	Q5HM04	rplV	fig|176279.9.peg.1783	J	50S ribosomal protein L22	8.05	21.03	26.78
SERP1827	Q5HM03	rpsS	fig|176279.9.peg.1784	J	30S ribosomal protein S19	19.81	14.13	13.71

SERP1828	Q5HM02	rplB	fig|176279.9.peg.1785	J	50S ribosomal protein L2	0.00	1000.00	1000.00
SERP1830	Q5HM00	rplD	fig|176279.9.peg.1787	J	50S ribosomal protein L4	0.00	1000.00	1000.00
SERP1831	Q5HLZ9	rplC	fig|176279.9.peg.1788	J	50S ribosomal protein L3	6.47	40.98	40.65
SERP1832	Q5HLZ8	rpsJ	fig|176279.9.peg.1789	J	30S ribosomal protein S10	2.53	4.45	15.72
SERP1841	Q5HLY9	SERP1841	fig|176279.9.peg.1796	-	Uncharacterized protein	0.00	0.00	1000.00
SERP1855	Q5HLX6	moeA	fig|176279.9.peg.1812	H	Molybdopterin molybdenumtransferase	-1000.00	-1000.00	-1000.00
SERP1857	Q5HLX4	moaB	fig|176279.9.peg.1814	H	Molybdenum cofactor biosynthesis protein B	0.00	0.00	1000.00
SERP1860	Q5HLX1	modB	fig|176279.9.peg.1817	P	Molybdenum transport system permease	-1000.00	-1000.00	-1000.00
SERP1861	Q5HLX0	modA	fig|176279.9.peg.1818	P	Molybdenum ABC transporter, molybdenum-binding protein ModA	2.46	3.82	3.63
SERP1869	Q5HLW3	ureA	fig|176279.9.peg.1826	E	Urease subunit gamma	-1000.00	3.44	3.03
SERP1870	Q5HLW2	ureB	fig|176279.9.peg.1827	E	Urease subunit beta	-1.53	-1.96	-4.50
SERP1872	Q5HLW0	ureE	fig|176279.9.peg.1829	O	Urease accessory protein UreE	3.02	2.07	-1.08
SERP1874	Q5HLV8	ureG	fig|176279.9.peg.1831	KO	Urease accessory protein UreG	-1000.00	-2.37	3.23
SERP1876	Q5HLV6	sarR	fig|176279.9.peg.1833	K	Staphylococcal accessory regulator R	1000.00	1000.00	1000.00
SERP1880	Q5HLV2	ssaA1	fig|176279.9.peg.1837	S	Staphylococcal secretory antigen SsaA	10.82	3.32	3.16
SERP1882	Q5HLV0	SERP1882	fig|176279.9.peg.1839	C	NAD/NADP octopine/nopaline dehydrogenase family protein	0.00	1000.00	0.00
SERP1883	Q5HLU9	SERP1883	fig|176279.9.peg.1840	S	Uncharacterized protein	-1000.00	2.99	4.70
SERP1888	Q5HLU4	SERP1888	fig|176279.9.peg.1844	CH	Putative 2-hydroxyacid dehydrogenase SERP1888	-39.82	-2.06	1.89
SERP1893	Q5HLT9	SERP1893	fig|176279.9.peg.1848	K	Transcriptional regulator, putative	-1.05	-1.55	-4.42
SERP1917	Q5HLR6	SERP1917	fig|176279.9.peg.1872	IQ	Oxidoreductase, short chain dehydrogenase/reductase family	-6.68	-2.45	-1.94
SERP1920	Q5HLR3	lyrA	fig|176279.9.peg.1875	S	Lysostaphin resistance protein A	1000.00	0.00	0.00
SERP1932	Q5HLQ2	SERP1932	fig|176279.9.peg.1883	S	DUF4097 domain-containing protein	0.00	1000.00	0.00
SERP1937	Q5HLP8	fni	fig|176279.9.peg.1887	C	Isopentenyl-diphosphate delta-isomerase	-7.95	5.07	6.22

SERP1942	Q5HLP3	SERP1942	fig|176279.9.peg.1892	-	Uncharacterized protein	-11.96	6.42	31.99
SERP1945	Q5HLP0	SERP1945	fig|176279.9.peg.1894	V	Drug transporter, putative	1.05	-2.04	1.27
SERP1948	Q5HLN7	tcaA	fig|176279.9.peg.1897	S	Membrane-associated protein TcaA	0.00	1000.00	1000.00
SERP1954	Q5HLN1	hssS	fig|176279.9.peg.1903	T	Heme sensor protein HssS	-1000.00	-4.06	-1000.00
SERP1959	Q5HLM6	SERP1959	fig|176279.9.peg.1908	-	Uncharacterized lipoprotein SERP1959	5.52	4.29	2.59
SERP1962	Q5HLM3	SERP1962	fig|176279.9.peg.1911	C	Alcohol dehydrogenase, zinc-containing	-1000.00	-1000.00	-1000.00
SERP1973	Q5HLL4	SERP1973	fig|176279.9.peg.1922	S	General stress protein 26, putative	-1.40	-1000.00	-1000.00
SERP1977	Q5HLL0	SERP1977	fig|176279.9.peg.1925	S	Uncharacterized protein	32.10	28.22	20.91
SERP1978	Q5HLK9	SERP1978	fig|176279.9.peg.1926	C	Nitroreductase family protein	0.00	0.00	1000.00
SERP1979	Q5HLK8	sarZ	fig|176279.9.peg.1927	K	HTH-type transcriptional regulator SarZ	0.00	0.00	1000.00
SERP1987	Q5HLK0	narG	fig|176279.9.peg.1935	C	Respiratory nitrate reductase, alpha subunit	0.00	1000.00	0.00
SERP1990	Q5HLJ7	nirB	fig|176279.9.peg.1938	C	Nitrite reductase [NAD(P)H], large subunit	0.00	0.00	1000.00
SERP1994	Q5HLJ3	SERP1994	fig|176279.9.peg.1940	E	ABC transporter, substrate-binding protein	0.00	0.00	1000.00
SERP2000	Q5HLI7	SERP2000	fig|176279.9.peg.1947	S	Lipoprotein, putative	11.30	8.57	2.24
SERP2005	Q5HLI2	SERP2005	fig|176279.9.peg.1953	ET	Amino acid ABC transporter, amino acid-binding protein	3.74	2.84	3.58
SERP2007	Q5HLI0	gpmA	fig|176279.9.peg.1955	G	2,3-bisphosphoglycerate-dependent phosphoglycerate mutase	-1000.00	-1000.00	-1000.00
SERP2013	Q5HLH4	SERP2013	fig|176279.9.peg.1961	S	Uncharacterized protein	1.36	7.83	5.67
SERP2022	Q5HLG5	SERP2022	fig|176279.9.peg.1969	H	2-dehydropantoate 2-reductase	-1000.00	-1000.00	-1000.00
SERP2029	Q5HLF8	SERP2029	fig|176279.9.peg.1976	M	Amino acid ABC transporter, amino acid-binding protein	0.00	1000.00	1000.00
SERP2031	Q5HLF6	SERP2031	fig|176279.9.peg.1978	P	Amino acid ABC transporter, ATP-binding protein	0.00	1000.00	1000.00
SERP2032	Q5HLF5	SERP2032	fig|176279.9.peg.1979	E	Sorbitol dehydrogenase, putative	-1000.00	-1000.00	-15.88
SERP2051	Q5HLD6	SERP2051	fig|176279.9.peg.1998	-	Uncharacterized protein	0.00	0.00	1000.00
SERP2053	Q5HLD4	SERP2053	fig|176279.9.peg.2000	L	MutT/nudix family protein	1.81	-1000.00	3.42

SERP2056	Q5HLD1	gtaB	fig|176279.9.peg.2003	M	UTP--glucose-1-phosphate uridylyltransferase	-2.61	1.31	-1.00
SERP2066	Q5HLC1	SERP2066	fig|176279.9.peg.2012	S	Uncharacterized protein	0.00	1000.00	1000.00
SERP2068	Q5HLB9	SERP2068	fig|176279.9.peg.2014	S	ABM domain-containing protein	-1.32	-4.88	-1000.00
SERP2069	Q5HLB8	SERP2069	fig|176279.9.peg.2015	G	Major facilitator superfamily protein	-1000.00	-7.73	-1000.00
SERP2079	Q5HLA8	SERP2079	fig|176279.9.peg.2024	S	Uncharacterized protein	-1000.00	-1000.00	-1000.00
SERP2082	Q5HLA5	SERP2082	fig|176279.9.peg.2027	S	Acetyltransferase, GNAT family	-1000.00	3.56	2.74
SERP2084	Q5HLA3	aldA	fig|176279.9.peg.2029	C	Putative aldehyde dehydrogenase AldA	-1000.00	-8.25	-1.17
SERP2086	Q5HLA1	SERP2086	fig|176279.9.peg.2031	C	Putative NAD(P)H nitroreductase SERP2086	0.00	0.00	1000.00
SERP2094	Q5HL93	sdhA	fig|176279.9.peg.2038	E	L-serine dehydratase	1.22	1.59	2.07
SERP2095	Q5HL92	sdhB	fig|176279.9.peg.2039	E	L-serine dehydratase	-1000.00	-1000.00	-1000.00
SERP2101	Q5HL86	rbsD	fig|176279.9.peg.2044	G	D-ribose pyranase	0.00	0.00	1000.00
SERP2102	Q5HL85	rbsU	fig|176279.9.peg.2045	U	Putative ribose uptake protein RbsU	0.00	0.00	1000.00
SERP2111	Q5HL76	SERP2111	fig|176279.9.peg.2054	CO	Thioredoxin, putative	-1000.00	-1000.00	5.60
SERP2112	Q5HL75	SERP2112	fig|176279.9.peg.2055	E	Alcohol dehydrogenase, zinc-containing	-1000.00	-21.10	-2.63
SERP2115	Q5HL72	SERP2115	fig|176279.9.peg.2058	C	Pyruvate oxidase	1000.00	0.00	1000.00
SERP2122	Q5HL65	SERP2122	fig|176279.9.peg.2065	I	Hydroxymethylglutaryl-CoA synthase	-1000.00	-1000.00	-1000.00
SERP2126	Q5HL61	feoB	fig|176279.9.peg.2069	P	Ferrous iron transport protein B	-1000.00	-1000.00	-1000.00
SERP2138	Q5HL49	isaA	fig|176279.9.peg.2080	M	Probable transglycosylase IsaA	3.37	2.94	1.39
SERP2145	Q5HL42	SERP2145	fig|176279.9.peg.2086	-	Uncharacterized protein	0.00	0.00	1000.00
SERP2148	Q5HL39	SERP2148	fig|176279.9.peg.2091	S	Uncharacterized protein	1000.00	0.00	1000.00
SERP2149	Q5HL38	SERP2149	fig|176279.9.peg.2092	M	Uncharacterized protein	0.00	1000.00	1000.00
SERP2150	Q5HL37	panD	fig|176279.9.peg.2093	H	Aspartate 1-decarboxylase	2.33	-1.25	-2.81
SERP2152	Q5HL35	panB	fig|176279.9.peg.2095	H	3-methyl-2-oxobutanoate hydroxymethyltransferase	0.00	1000.00	1000.00
SERP2154	Q5HL33	budA	fig|176279.9.peg.2097	Q	Alpha-acetolactate decarboxylase	-56.95	-12.81	-3.90
SERP2155	Q5HL32	budB	fig|176279.9.peg.2098	EH	Acetolactate synthase, catabolic	-3.86	-5.42	-3.93
SERP2156	Q5HL31	ldh	fig|176279.9.peg.2099	C	L-lactate dehydrogenase	-10.03	-6.05	-2.89
SERP2161	Q5HL26	SERP2161	fig|176279.9.peg.2104	-	Lipoprotein, putative	1000.00	1000.00	1000.00

SERP2166	Q5HL21	fda	fig|176279.9.peg.2109	G	Fructose-bisphosphate aldolase class 1	-1.41	-1.70	-2.90
SERP2167	Q5HL20	SERP2167	fig|176279.9.peg.2110	-	Lipoprotein, putative	3.47	19.91	27.49
SERP2168	Q5HL19	mqo4	fig|176279.9.peg.2111	C	Probable malate:quinone oxidoreductase 4	1.40	1.59	3.64
SERP2174	Q5HL13	SERP2174	fig|176279.9.peg.2116	S	YozE_SAM_like domain-containing protein	0.00	0.00	1000.00
SERP2176	Q5HL11	betA	fig|176279.9.peg.2118	E	Oxygen-dependent choline dehydrogenase	-1000.00	-1000.00	-1000.00
SERP2177	Q5HL10	betB	fig|176279.9.peg.2119	C	Betaine aldehyde dehydrogenase	-1000.00	-1000.00	-2.99
SERP2178	Q5HL09	SERP2178	fig|176279.9.peg.2120	K	HTH-type transcriptional regulator	-1000.00	30.61	2.92
SERP2186	Q5HL01	sat	fig|176279.9.peg.2128	H	Sulfate adenylyltransferase	-1000.00	-1000.00	-1000.00
SERP2187	Q5HL00	SERP2187	fig|176279.9.peg.2129	S	Probable membrane transporter protein	-1000.00	16.72	16.88
SERP2191	Q5HKZ6	cysJ	fig|176279.9.peg.2133	C	Sulfite reductase [NADPH] flavoprotein alpha-component	0.00	0.00	1000.00
SERP2199	Q5HKY8	SERP2199	fig|176279.9.peg.2140	S	Uncharacterized protein	-1000.00	-1000.00	-1000.00
SERP2202	Q5HKY5	SERP2202	fig|176279.9.peg.2143	S	DUF4064 domain-containing protein	37.00	18.95	7.20
SERP2207	Q5HKY0	SERP2207	fig|176279.9.peg.2148	-	Uncharacterized protein	-1000.00	-1000.00	-6.81
SERP2211	Q5HKX6	SERP2211	fig|176279.9.peg.2152	S	Uncharacterized protein	1000.00	0.00	0.00
SERP2225	Q5HKW3	SERP2225	fig|176279.9.peg.2166	K	Transcriptional regulator, ArsR family	0.00	0.00	1000.00
SERP2242	Q5HKV0	ipdC	fig|176279.9.peg.2180	GH	Indole-3-pyruvate decarboxylase	0.00	1000.00	1000.00
SERP2250	Q5HKU2	arcA	fig|176279.9.peg.2188	E	Arginine deiminase	-1000.00	1.92	14.22
SERP2252	Q5HKU0	sepA	fig|176279.9.peg.2190	E	Neutral metalloproteinase	1000.00	1000.00	1000.00
SERP2257	Q5HKT5	SERP2257	fig|176279.9.peg.2195	IQ	Oxidoreductase, short-chain dehydrogenase/reductase family	-3.72	-2.96	-2.24
SERP2262	Q5HKT0	SERP2262	fig|176279.9.peg.2200	GM	Phage infection protein	1000.00	1000.00	1000.00
SERP2263	Q5HKS9	SERP2263	fig|176279.9.peg.2201	NU	N-acetylmuramoyl-L-alanine amidase domain protein	3.01	1.49	-5.40
SERP2264	Q5HKS8	SERP2264	fig|176279.9.peg.2202	-	Cell wall surface anchor family protein	8.87	16.45	1.69

SERP2265	Q5HKS7	SERP2265	fig|176279.9.peg.2203	O	Organic hydroperoxide resistance protein-like 2	2.21	-3.63	-1.94
SERP2273	Q5HKR9	SERP2273	fig|176279.9.peg.2210	-	Uncharacterized protein	-1000.00	-1000.00	-1.53
SERP2281	Q5HKR2	SERP2281	fig|176279.9.peg.2218	N	Serine threonine rich antigen	6.07	2.14	-4.51
SERP2286	Q5HKQ7	SERP2286	fig|176279.9.peg.2223	P	Phosphonate ABC transporter, phosphonate-binding protein	6.26	10.52	7.04
SERP2287	Q5HKQ6	SERP2287	fig|176279.9.peg.2224	S	Uncharacterized protein	57.84	46.30	75.80
SERP2295	Q5HKP8	icaB	fig|176279.9.peg.2230	G	Poly-beta-1,6-N-acetyl-D-glucosamine N-deacetylase	1000.00	1000.00	1000.00
SERP2297	Q5HKP6	lip	fig|176279.9.peg.2232	D	Lipase	-1000.00	-1000.00	-1000.00
SERP2312	Q5HKN2	mqo2	fig|176279.9.peg.2246	C	Probable malate:quinone oxidoreductase 2	1.18	1.69	3.07
SERP2314	Q5HKN0	SERP2314	fig|176279.9.peg.2247	S	UPF0312 protein SERP2314	-6.70	-5.82	-6.20
SERP2320	Q5HKM4	SERP2320	fig|176279.9.peg.2253	M	Choline/carnitine/betaine transporter	-1000.00	-1000.00	-1000.00
SERP2324	Q5HKM0	SERP2324	fig|176279.9.peg.2257	C	Dihydrolipoamide acetyltransferase component of pyruvate dehydrogenase complex	-1000.00	-1000.00	-4.55
SERP2325	Q5HKL9	SERP2325	fig|176279.9.peg.2258	C	Acetoin dehydrogenase, E1 component, beta subunit	-1000.00	-1000.00	-1000.00
SERP2333	Q5HKL1	SERP2333	fig|176279.9.peg.2265	S	Uncharacterized protein	-1000.00	-1000.00	-1000.00
SERP2340	Q5HKK4	SERP2340	fig|176279.9.peg.2271	EGP	Drug resistance transporter, EmrB/QacA family	-1000.00	-1000.00	-1000.00
SERP2343	Q5HKK1	SERP2343	fig|176279.9.peg.2274	S	PTS EIIA type-4 domain-containing protein	-2.27	-2.19	-3.46
SERP2345	Q5HKJ9	SERP2345	fig|176279.9.peg.2276	G	Dihydroxyacetone kinase family protein	-1000.00	-4.11	2.57
SERP2346	Q5HKJ8	gldA	fig|176279.9.peg.2277	C	Glycerol dehydrogenase	-1000.00	-1000.00	-1000.00
SERP2347	Q5HKJ7	bioB	fig|176279.9.peg.2278	H	Biotin synthase	0.00	1000.00	0.00
SERP2350	Q5HKJ5	SERP2350	fig|176279.9.peg.2282	-	Uncharacterized protein	1.64	-7.05	-1.61
SERP2351	Q5HKJ4	arcB-2	fig|176279.9.peg.2283	E	Ornithine carbamoyltransferase	-10.89	-1000.00	-1000.00
SERP2352	Q5HKJ3	arcC	fig|176279.9.peg.2284	E	Carbamate kinase	-4.17	-13.57	-11.81
SERP2362	Q5HKI3	SERP2362	fig|176279.9.peg.2294	K	Transcriptional regulator, TetR family	1000.00	0.00	0.00

SERP2365	Q5HKI0	pflA	fig|176279.9.peg.2297	H	Pyruvate formate-lyase-activating enzyme	0.00	0.00	1000.00
SERP2366	Q5HKH9	pflB	fig|176279.9.peg.2298	C	Formate acetyltransferase	-1000.00	-1.57	1.03
SERP2379	Q5HKG6	butA	fig|176279.9.peg.2310	IQ	Diacetyl reductase [(S)-acetoin forming]	-6.94	-2.19	-1.50
SERP2381	Q5HKG4	SERP2381	fig|176279.9.peg.2312	C	NADH:flavin oxidoreductase/fumarate reductase, flavoprotein subunit, putative	-1000.00	-1000.00	3.17
SERP2383	Q5HKG2	SERP2383	fig|176279.9.peg.2314	P	ABC transporter, substrate-binding protein	5.15	107.65	83.86
SERP2388	Q5HKF8	geh-2	fig|176279.9.peg.2320	D	Lipase	15.08	108.01	7.98
SERP2392	Q5HKF4	bhp	fig|176279.9.peg.2323	O	Cell wall associated biofilm protein	-7.05	-52.76	3.08
SERP2394	Q5HKF2	SERP2394	fig|176279.9.peg.2325	E	Aminotransferase, class II	-1000.00	-1000.00	-1000.00
SERP2398	Q5HKE8	aap	fig|176279.9.peg.2329	DM	Accumulation associated protein	5.97	1.82	-1.73
SERP2411	Q5HKD7	SERP2411	fig|176279.9.peg.2341	IQ	Oxidoreductase, short chain dehydrogenase/reductase family	-18.46	-2.05	-2.52
SERP2412	Q5HKD6	mqo3	fig|176279.9.peg.2342	C	Probable malate:quinone oxidoreductase 3	-1000.00	-3.40	6.89
SERP2417	Q5HKD1	SERP2417	fig|176279.9.peg.2345	-	Lipoprotein, putative	2.52	1.25	1.40
SERP2427	Q5HKC1	arsD	fig|176279.9.peg.2355	S	Arsenical resistance operon trans-acting repressor	-1.02	14.56	-3.16
SERP2431	Q5HKB7	arsC2	fig|176279.9.peg.2359	T	Arsenate reductase 2	1.40	-4.24	-1000.00
SERP2437	Q5HKB1	SERP2437	fig|176279.9.peg.2364	M	Lipoprotein, putative	-1000.00	5.84	11.37
SERP2459	Q5HK91	SERP2459	fig|176279.9.peg.2384	L	CRISPR-associated protein, TM1792 family	-1000.00	-1000.00	3.62
SERP2463	Q5HK87	cas1	fig|176279.9.peg.2388	L	CRISPR-associated endonuclease Cas1	0.00	1000.00	0.00
SERP2472	Q5HK78	hsdM	fig|176279.9.peg.2396	V	Type I restriction-modification system, M subunit	-1000.00	-1000.00	5.44
SERP2485	Q5HK65	kdpC	fig|176279.9.peg.2409	P	Potassium-transporting ATPase KdpC subunit	0.00	0.00	1000.00
SERP2521	Q5HK31	mecA	fig|176279.9.peg.2441	M	Penicillin-binding protein 2	0.00	0.00	1000.00
SERP2534	Q5HK18	walR	fig|176279.9.peg.2453	K	Transcriptional regulatory protein WalR	-1000.00	17.76	-1000.00
SERP2537	Q5HK15	dnaB	fig|176279.9.peg.2455	L	Replicative DNA helicase	-1.21	-2.55	-92.89

SERP2545	Q5HK07	serS	fig|176279.9.peg.2463	J	Serine--tRNA ligase	-3.87	-2.06	-1.08
SERP2551	Q5HK01	SERP2551	fig|176279.9.peg.2468	S	S4 RNA-binding domain-containing protein	-3.85	1.14	1.72
SERP2552	Q5HK00	dnaN	fig|176279.9.peg.2469	L	Beta sliding clamp	-1000.00	-10.91	-1.07

Table. S2. KEGG pathways of up-regulated proteins

General description	Functional category	KEGG pathway	Gene count		
			T1	T2	T3
Metabolism	Global and overview maps	01100 Metabolic pathways	18	39	84
		01110 Biosynthesis of secondary metabolites	6	14	40
		01120 Microbial metabolism in diverse environments	4	9	30
		01200 Carbon metabolism	2	6	22
		01210 2-Oxocarboxylic acid metabolism	0	0	3
		01212 Fatty acid metabolism	1	1	4
		01230 Biosynthesis of amino acids	1	3	12
		01250 Biosynthesis of nucleotide sugars	0	0	4
		01240 Biosynthesis of cofactors	2	10	16
		01220 Degradation of aromatic compounds	1	0	0
	Amino acid metabolism	00250 Alanine, aspartate and glutamate metabolism	0	1	5
		00260 Glycine, serine and threonine metabolism	0	1	4
		00270 Cysteine and methionine metabolism	0	1	3
		00280 Valine, leucine and isoleucine degradation	0	0	3
		00290 Valine, leucine and isoleucine biosynthesis	0	0	0
		00300 Lysine biosynthesis	1	1	3
		00310 Lysine degradation	0	0	2
		00220 Arginine biosynthesis	0	1	2
		00330 Arginine and proline metabolism	0	0	2
		00340 Histidine metabolism	0	0	2
		00350 Tyrosine metabolism	0	0	0
		00360 Phenylalanine metabolism	0	0	0
		00380 Tryptophan metabolism	1	0	2
		00400 Phenylalanine, tyrosine and tryptophan biosynthesis	0	1	1
		00261 Monobactam biosynthesis	0	0	2

Biosynthesis of other secondary metabolites	00521 Streptomycin biosynthesis	1	0	1
	00401 Novobiocin biosynthesis	0	0	0
	00999 Biosynthesis of various plant secondary metabolites	0	1	1
	00997 Biosynthesis of various other secondary metabolites	1	0	0
Carbohydrate metabolism	00010 Glycolysis / Gluconeogenesis	0	0	4
	00020 Citrate cycle (TCA cycle)	0	2	11
	00030 Pentose phosphate pathway	0	0	3
	00040 Pentose and glucuronate interconversions	0	0	1
	00051 Fructose and mannose metabolism	1	1	1
	00052 Galactose metabolism	0	0	1
	00500 Starch and sucrose metabolism	0	0	1
	00520 Amino sugar and nucleotide sugar metabolism	0	0	4
	00620 Pyruvate metabolism	2	5	14
	00630 Glyoxylate and dicarboxylate metabolism	1	2	3
	00640 Propanoate metabolism	1	2	8
	00650 Butanoate metabolism	0	1	1
	00660 C5-Branched dibasic acid metabolism	0	0	2
	00562 Inositol phosphate metabolism	1	0	0
Energy metabolism	00190 Oxidative phosphorylation	3	5	7
	00680 Methane metabolism	0	1	1
	00910 Nitrogen metabolism	0	1	2
	00920 Sulfur metabolism	0	0	1
Glycan biosynthesis and metabolism	00542 O-Antigen repeat unit biosynthesis	0	0	0
	00541 O-Antigen nucleotide sugar biosynthesis	0	0	0
	00550 Peptidoglycan biosynthesis	1	3	5
Lipid metabolism	00061 Fatty acid biosynthesis	1	1	4
	00071 Fatty acid degradation	0	0	1
	00561 Glycerolipid metabolism	2	3	6
	00564 Glycerophospholipid metabolism	0	0	1

		00590 Arachidonic acid metabolism	0	0	0
	Metabolism of cofactors and vitamins	00730 Thiamine metabolism	1	1	1
		00740 Riboflavin metabolism	0	0	0
		00750 Vitamin B6 metabolism	0	0	0
		00760 Nicotinate and nicotinamide metabolism	0	1	2
		00770 Pantothenate and CoA biosynthesis	1	1	2
		00780 Biotin metabolism	0	1	0
		00785 Lipoic acid metabolism	0	1	1
		00790 Folate biosynthesis	0	1	3
		00670 One carbon pool by folate	0	0	1
		00860 Porphyrin metabolism	0	0	1
		00130 Ubiquinone and other terpenoid-quinone biosynthesis	0	1	1
	Metabolism of other amino acids	00410 beta-Alanine metabolism	1	0	1
		00430 Taurine and hypotaurine metabolism	0	1	2
		00450 Selenocompound metabolism	0	1	1
		00460 Cyanoamino acid metabolism	0	0	0
		00470 D-Amino acid metabolism	0	1	0
		00480 Glutathione metabolism	0	0	1
	Metabolism of terpenoids and polyketides	00900 Terpenoid backbone biosynthesis	0	1	2
	Nucleotide metabolism	00230 Purine metabolism	1	4	7
		00240 Pyrimidine metabolism	1	2	6
	Xenobiotics biodegradation and metabolism	00362 Benzoate degradation	1	0	0
		00625 Chloroalkane and chloroalkene degradation	0	0	1
		00622 Xylene degradation	1	0	0
		00626 Naphthalene degradation	0	0	0
Cellular Processes	Cellular community - prokaryotes	02024 Quorum sensing	4	6	6
	Cell motility	02040 Flagellar assembly	2	2	2
Environmental Information Processing	Membrane transport	02010 ABC transporters	7	9	10
		02060 Phosphotransferase system (PTS)	1	1	1
		03070 Bacterial secretion system	1	3	4

	Signal transduction	02020 Two-component system	0	4	4
Genetic Information Processing	Transcription	03020 RNA polymerase	2	4	4
	Translation	03010 Ribosome	27	35	37
		00970 Aminoacyl-tRNA biosynthesis	1	5	10
	Folding, sorting and degradation	03060 Protein export	2	5	6
		04122 Sulfur relay system	0	0	1
		03018 RNA degradation	1	2	5
	Replication and repair	03030 DNA replication	0	0	1
		03410 Base excision repair	0	1	1
		03420 Nucleotide excision repair	0	0	0
		03430 Mismatch repair	1	0	1
		03440 Homologous recombination	0	0	2
Human Diseases	Drug resistance: antimicrobial	01501 beta-Lactam resistance	1	3	4
		01502 Vancomycin resistance	0	0	0
		01503 Cationic antimicrobial peptide (CAMP) resistance	1	2	3

Table. S3. KEGG pathways of down-regulated proteins

General description	Functional category	KEGG pathway	Gene count T1	T2	T3
Metabolism	Global and overview maps	01100 Metabolic pathways	95	65	43
		01110 Biosynthesis of secondary metabolites	62	43	30
		01120 Microbial metabolism in diverse environments	39	24	14
		01200 Carbon metabolism	28	15	8
		01210 2-Oxocarboxylic acid metabolism	6	4	4
		01212 Fatty acid metabolism	4	1	1
		01230 Biosynthesis of amino acids	21	14	10
		01250 Biosynthesis of nucleotide sugars	4	2	0
		01240 Biosynthesis of cofactors	19	12	7
		01220 Degradation of aromatic compounds	3	3	3
	Amino acid metabolism	00250 Alanine, aspartate and glutamate metabolism	4	4	2
		00260 Glycine, serine and threonine metabolism	8	7	7
		00270 Cysteine and methionine metabolism	6	6	4
		00280 Valine, leucine and isoleucine degradation	5	3	1
		00290 Valine, leucine and isoleucine biosynthesis	1	1	1
		00300 Lysine biosynthesis	2	2	2
		00310 Lysine degradation	5	2	2
		00220 Arginine biosynthesis	8	6	6
		00330 Arginine and proline metabolism	3	2	2
		00340 Histidine metabolism	2	2	0
		00350 Tyrosine metabolism	3	3	3
		00360 Phenylalanine metabolism	1	0	1
		00380 Tryptophan metabolism	4	2	1
		00400 Phenylalanine, tyrosine and tryptophan biosynthesis	1	0	0
	Biosynthesis of other secondary metabolites	00261 Monobactam biosynthesis	3	3	3
		00521 Streptomycin biosynthesis	1	1	0

	00401 Novobiocin biosynthesis	0	0	0
	00999 Biosynthesis of various plant secondary metabolites	0	0	0
	00997 Biosynthesis of various other secondary metabolites	0	0	0
Carbohydrate metabolism	00010 Glycolysis / Gluconeogenesis	15	13	7
	00020 Citrate cycle (TCA cycle)	11	5	2
	00030 Pentose phosphate pathway	7	6	2
	00040 Pentose and glucuronate interconversions	1	0	0
	00051 Fructose and mannose metabolism	2	2	1
	00052 Galactose metabolism	3	3	0
	00500 Starch and sucrose metabolism	3	2	0
	00520 Amino sugar and nucleotide sugar metabolism	5	3	1
	00620 Pyruvate metabolism	14	10	6
	00630 Glyoxylate and dicarboxylate metabolism	5	2	1
	00640 Propanoate metabolism	9	5	3
	00650 Butanoate metabolism	11	8	7
	00660 C5-Branched dibasic acid metabolism	4	3	2
	00562 Inositol phosphate metabolism	0	0	0
Energy metabolism	00190 Oxidative phosphorylation	3	1	0
	00680 Methane metabolism	5	2	2
	00910 Nitrogen metabolism	2	2	1
	00920 Sulfur metabolism	2	1	1
Glycan biosynthesis and metabolism	00542 O-Antigen repeat unit biosynthesis	0	0	0
	00541 O-Antigen nucleotide sugar biosynthesis	1	0	0
	00550 Peptidoglycan biosynthesis	1	1	1
Lipid metabolism	00061 Fatty acid biosynthesis	3	1	1
	00071 Fatty acid degradation	5	4	3
	00561 Glycerolipid metabolism	4	4	2
	00564 Glycerophospholipid metabolism	0	0	0
	00590 Arachidonic acid metabolism	0	0	1

	Metabolism of cofactors and vitamins	00730 Thiamine metabolism	0	0	0
		00740 Riboflavin metabolism	1	1	1
		00750 Vitamin B6 metabolism	2	1	0
		00760 Nicotinate and nicotinamide metabolism	1	0	0
		00770 Pantothenate and CoA biosynthesis	4	3	3
		00780 Biotin metabolism	2	1	1
		00785 Lipoic acid metabolism	1	0	0
		00790 Folate biosynthesis	1	1	1
		00670 One carbon pool by folate	1	1	1
		00860 Porphyrin metabolism	2	2	0
		00130 Ubiquinone and other terpenoid-quinone biosynthesis	1	1	1
	Metabolism of other amino acids	00410 beta-Alanine metabolism	1	1	1
		00430 Taurine and hypotaurine metabolism	2	1	0
		00450 Selenocompound metabolism	3	2	2
		00460 Cyanoamino acid metabolism	0	0	0
		00470 D-Amino acid metabolism	4	3	3
		00480 Glutathione metabolism	2	1	1
	Metabolism of terpenoids and polyketides	00900 Terpenoid backbone biosynthesis	4	2	1
	Nucleotide metabolism	00230 Purine metabolism	10	8	7
		00240 Pyrimidine metabolism	5	3	2
	Xenobiotics biodegradation and metabolism	00362 Benzoate degradation	1	0	0
		00625 Chloroalkane and chloroalkene degradation	4	4	3
		00622 Xylene degradation	0	0	0
		00626 Naphthalene degradation	3	3	3
Cellular Processes	Cellular community - prokaryotes	02024 Quorum sensing	3	2	1
	Cell motility	02040 Flagellar assembly	0	0	0
Environmental Information Processing	Membrane transport	02010 ABC transporters	2	3	4
		02060 Phosphotransferase system (PTS)	3	5	3
		03070 Bacterial secretion system	2	1	1

	Signal transduction	02020	Two-component system	7	6	3
Genetic Information Processing	Transcription	03020	RNA polymerase	0	0	0
	Translation	03010	Ribosome	5	2	0
		00970	Aminoacyl-tRNA biosynthesis	9	7	3
	Folding, sorting and degradation	03060	Protein export	2	1	1
		04122	Sulfur relay system	0	0	0
		03018	RNA degradation	3	2	1
	Replication and repair	03030	DNA replication	3	4	3
		03410	Base excision repair	0	0	0
		03420	Nucleotide excision repair	2	2	2
		03430	Mismatch repair	3	4	2
		03440	Homologous recombination	3	3	2
Human Diseases	Drug resistance: antimicrobial	01501	beta-Lactam resistance	0	0	0
		01502	Vancomycin resistance	1	1	1
		01503	Cationic antimicrobial peptide (CAMP) resistance	1	1	0

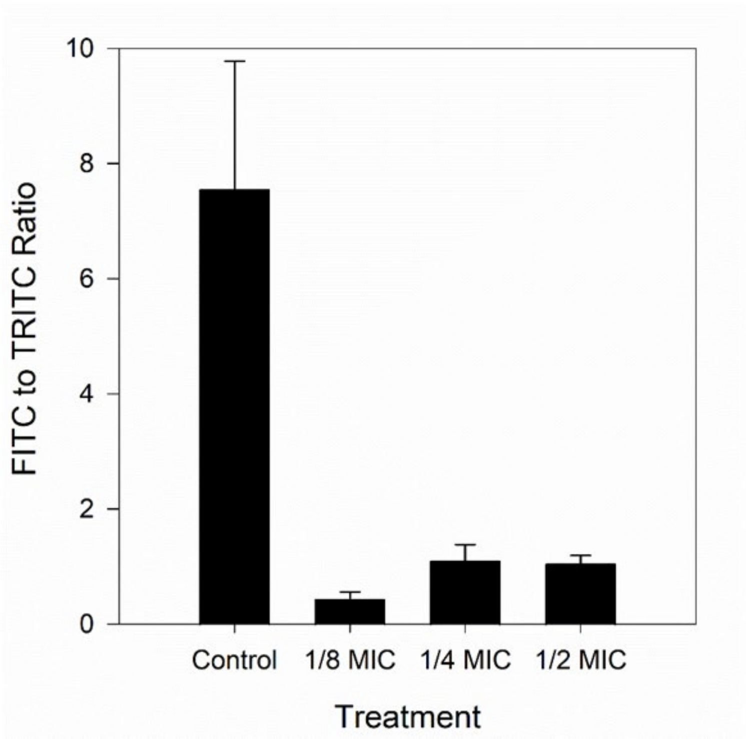

Supplemental Figure S1. The ratio of FITC (live cells) to TRITC (dead cells) in biofilms. Biofilm images were acquired using CLSM and the ratio of FITC (live cells) to TRITC (dead cells) was analyzed via one-way ANOVA. One experiment was performed in which z-stack images were collected using a Plan Flour 40x (6 images per treatment group, 20 µm thickness, 0.5 µm slices). While there was a significant between-group effect ($p<0.001$), there were no differences among groups that received TC (1/8, 1/4, or 1/2 MIC; multiple comparison tests, all *p*'s>0.05). Thus, in this imaging experiment, TC concentration had no effect on the number of dead cells in the biofilms.
